# Dysfunction of Glutamate Delta-1 Receptor-Cerebellin 1 Trans-Synaptic Signaling in the Central Amygdala in Chronic Pain

**DOI:** 10.3390/cells10102644

**Published:** 2021-10-03

**Authors:** Pauravi J. Gandhi, Dinesh Y. Gawande, Gajanan P. Shelkar, Sukanya G. Gakare, Takaki Kiritoshi, Guangchen Ji, Bishal Misra, Ratnamala Pavuluri, Jinxu Liu, Volker Neugebauer, Shashank M. Dravid

**Affiliations:** 1 Department of Pharmacology and Neuroscience, Creighton University School of Medicine, Omaha, NE 68178, USA; pauravigandhi@creighton.edu (P.J.G.); dineshgawande@creighton.edu (D.Y.G.); gajananshelkar@creighton.edu (G.P.S.); sukanyagakare@creighton.edu (S.G.G.); BishalMisra@creighton.edu (B.M.); rup50341@creighton.edu (R.P.); jinxuliu@hotmail.com (J.L.); 2 Department of Pharmacology and Neuroscience, School of Medicine, Texas Tech University Health Sciences Center, Lubbock, TX 79430, USA; Takaki.Kiritoshi@ttuhsc.edu (T.K.); Guangchen.Ji@ttuhsc.edu (G.J.); Volker.Neugebauer@ttuhsc.edu (V.N.); 3 Center of Excellence for Translational Neuroscience and Therapeutics, Texas Tech University Health Sciences Center, Lubbock, TX 79430, USA; 4 Garrison Institute on Aging, Texas Tech University Health Sciences Center, Lubbock, TX 79430, USA

**Keywords:** GluD1, Cbln1, amygdala, pain, parabrachial nucleus, CGRP, PKCdelta, glutamate

## Abstract

Chronic pain is a debilitating condition involving neuronal dysfunction, but the synaptic mechanisms underlying the persistence of pain are still poorly understood. We found that the synaptic organizer glutamate delta 1 receptor (GluD1) is expressed postsynaptically at parabrachio-central laterocapsular amygdala (PB-CeLC) glutamatergic synapses at axo-somatic and punctate locations on protein kinase C δ -positive (PKCδ^+^) neurons. Deletion of GluD1 impairs excitatory neurotransmission at the PB-CeLC synapses. In inflammatory and neuropathic pain models, GluD1 and its partner cerebellin 1 (Cbln1) are downregulated while AMPA receptor is upregulated. A single infusion of recombinant Cbln1 into the central amygdala led to sustained mitigation of behavioral pain parameters and normalized hyperexcitability of central amygdala neurons. Cbln2 was ineffective under these conditions and the effect of Cbln1 was antagonized by GluD1 ligand D-serine. The behavioral effect of Cbln1 was GluD1-dependent and showed lateralization to the right central amygdala. Selective ablation of GluD1 from the central amygdala or injection of Cbln1 into the central amygdala in normal animals led to changes in averse and fear-learning behaviors. Thus, GluD1-Cbln1 signaling in the central amygdala is a teaching signal for aversive behavior but its sustained dysregulation underlies persistence of pain. Significance statement: Chronic pain is a debilitating condition which involves synaptic dysfunction, but the underlying mechanisms are not fully understood. Our studies identify a novel mechanism involving structural synaptic changes in the amygdala caused by impaired GluD1-Cbln1 signaling in inflammatory and neuropathic pain behaviors. We also identify a novel means to mitigate pain in these conditions using protein therapeutics.

## 1. Introduction

Pain perception is essential for survival; however, chronic pain can produce long-term disability. The major pain pathways include the spinothalamic and spino-parabrachio-amygdaloid pathways [1]. In the spino-parabrachio-amygdaloid pathway, glutamatergic neurons in the external lateral parabrachial nucleus (PB) project to the lateral and capsular divisions of the central amygdala (CeLC), which have been termed the “nociceptive amygdala” [1,2,3,4,5,6]. Studies have established functional plasticity at the parabrachio-amygdala synapses in the CeA as a mechanism for persistent and chronic pain [6,7,8,9,10,11,12,13,14,15]. Increases in PB-CeLC neurotransmission and CeLC neuron hyperexcitability are well documented in inflammatory [7,8,9,10,11,13,14,16,17,18,19,20] and neuropathic pain models [11,21,22]. Recent studies have also begun to analyze the CeLC cell type–specific role in pain circuitry and pain processing. Two major non-overlapping cell populations are defined by their expression of protein kinase C δ (PKCδ) or somatostatin (SOM). Evidence suggests that PKCδ^+^ neurons are the main targets of synapses from PB-calcitonin gene-related peptide (CGRP) terminals [23,24,25,26,27], while SOM^+^ neurons receive inputs from primarily CGRP-negative PB cells [28,29,30]. SOM^+^ neurons are more excitable and have more complex dendritic arborization compared to PKCδ^+^ neurons [31]. In a neuropathic pain model hyperexcitability was noted specifically in the CeLC PKCδ^+^ neuron subtype [29]. An increase in synaptic efficacy of presumable PKCδ^+^ neurons was also observed in a neuropathic pain model [28]. In contrast, no change in neuronal excitability [29], but a decrease in synaptic efficacy [28] was found in CeLC SOM^+^ neurons in neuropathic pain models. Importantly, chemogenetic manipulation studies under normal conditions and in the neuropathic pain model suggests that PKCδ^+^ neurons are a pain generating cell-type while SOM^+^ are a pain reducing cell-type [29]. Despite these important advances, a significant knowledge gap remains in our understanding of the structural synaptic signaling under normal conditions and in pain conditions associated with dysfunction of PB-CeLC neurotransmission.

GluD1 and GluD2 form the delta family of ionotropic GluRs (iGluRs). However, GluDs are unusual because they do not exhibit typical agonist-induced currents like other iGluRs [32,33]. Instead, GluDs are involved in synapse formation and maintenance through interactions with presynaptic Neurexin and intermediary protein Cbln1 [34,35,36,37,38,39]. Together, the GluD-Cbln1-Neurexin forms a trans-synaptic complex that regulates synapse formation and maintenance. Recently, a role of GluD1 has been established at thalamostriatal synapses as well as hippocampal synapses [40,41]. Neuroanatomical studies have found strong expression of GluD1 protein in the CeA [42,43], but this region does not show Cbln1, Cbln2, or Cbln4 mRNA expression [44,45]. However, Cbln1 mRNA is heavily expressed in PB neurons [44,45], suggesting that the GluD1-Cbln1 complex may contribute to PB-CeLC glutamatergic synapses. Furthermore, GluD1 KO and Cbln1 KO mice display impaired fear acquisition and altered anxiety-like behaviors [45,46], which are amygdala-dependent behaviors. The specific role of GluD1-Cbln1 structural signaling in regulating PB-CeLC synapses and pain-related behaviors is unknown. In the present study, we tested the hypothesis that a transsynaptic GluD1-Cbln1 complex is a novel substrate for pain persistence. Our findings demonstrate that this complex undergoes aberrant changes in pain states and rescue of GluD1-Cbln1 signaling mitigates pain behaviors by restoring synaptic function.

## 2. Material and Methods

### 2.1. Animals

Wildtype and GluD1 KO male and female mice were group housed in the animal house facility at a constant temperature (22 ± 1 °C) and a 12 hr light–dark cycle with free access to food and water. Behavioral testing was performed between 9:00 a.m. and 4:00 p.m. GluD1 KO mice were obtained from Dr. Jian Zuo [47]. These were on >95% C57BL/6 and remaining 129SvEv background. GluD1^flox/flox^ mice on congenic C57BL/6 background were obtained from Dr. Pei Lung-Chen with loxP sites in intron 10 and 12. PKCδ Cre mice (on congenic C57BL/6 background) were obtained from Dr. David Anderson with GluClα-ires-Cre cassette inserted into the PKCδ gene. In naïve animals (without any surgical manipulation or pain induction), electrophysiology and immunohistochemical studies were carried out at 4–6 weeks of age and 2–3 months of age for behavioral studies. For mice with surgical manipulation or pain induction, all studies were carried out at 3–4 months of age to correlate changes in synapse structure and function with pain behaviors. In this study, strict measures were taken to minimize pain and suffering to animals in accordance with the recommendations in the Guide for Care and Use of Laboratory Animals of the National Institutes of Health. All experimental protocols were approved by the Creighton University Institutional Animal Care and Use Committee Policies and Procedures.

Adult Sprague–Dawley rats, 250 g to 350 g at time of testing, were housed 3 per cage on a 12 h light–dark cycle (lights on at 7:00 a.m.) with unrestricted access to food and water. On the day of the experiment, animals were transferred from the animal facility to the laboratory and acclimated for at least 1 h. All procedures were approved by the Institutional Animal Care and Use Committees (IACUC) of Texas Tech University Health Sciences Center. All studies were conducted in accordance with the policies and recommendations of the National Institutes of Health Guide for the Care and Use of Laboratory Animals. Experiments were done in a blinded fashion and reproduced by several investigators.

### 2.2. Immunohistochemistry

Mice or rats were transcardially perfused with 4% PFA in 0.1 M phosphate buffer (PB) pH 7.4, and brains collected and incubated overnight in the same fixative at 4 °C. Brains were then transferred successively into solutions of 10%, 20%, and 30% sucrose in 0.1 M PB and thereafter frozen at −30 °C to −40 °C using isopentane. For immunohistochemistry, 20-μm-thick coronal sections were cut using a cryostat (Leica CM 1900). After washing, sections were incubated in blocking solution containing 10% normal goat serum (Jackson ImmunoResearch Laboratories Inc., West Grove, PA, USA, Catalog#005-000-121) or chicken serum (Jackson ImmunoResearch Laboratories Inc., Catalog#003-000-120) or normal donkey serum in 0.25% Triton-X in 0.1 M PB (PBT) for 1 h at room temperature. Following blocking, sections were incubated overnight at 4 °C in primary antibodies at appropriate concentrations in PBT guinea pig GluD1 primary antibody (1:500, GluD1C-GP-Af860, Frontier Institute Co., Ltd., Sapporo, Japan), rabbit vGluT1 (1:10000, VGT1-3, MAb technologies, Stone Mountain, GA, USA), chicken vGluT2 (1:1000, 135416, Synaptic systems, Goettingen, Germany), rabbit CGRP (1:500, PC205L, Millipore Sigma, Burlington, MA, USA), mouse PKCδ (1:500, # 10397, BD Biosciences, San Jose, CA, USA), rabbit SST (1:500, # T-4103, Peninsula Laboratories, BMA Biomedicals, Augst, Switzerland), rabbit PSD93 (1:500, #AF 769, Frontier Institute Co., Ltd.), or rabbit PSD95 (1:500, # 51-690, Invitrogen, Carlsbad, CA, USA). For primary antibodies rabbit Cbln1 (1:500, Cbln1-Rb-Af270, Frontier Institute Co., Ltd.) and GluA1 (1:500, GluA1-Rb-Af690, Frontier Institute Co., Ltd.), 100 μm thick coronal sections were cut and epitope retrieval was performed using pepsin and citrate treatment, respectively, before blocking. For pepsin treatment, sections were incubated for 3 min in 1 mg/mL pepsin in 0.9 N HCl at 37 °C. For citrate treatment, sections were incubated in sodium citrate buffer (10 mM sodium citrate, 0.05% Tween 20, pH 6.0) at 90 °C for 20 min. The day after incubation with primary antibody, sections were washed and thereafter incubated with the appropriate secondary antibodies; goat anti-guinea pig conjugated to AlexaFluor 488 (1:500, A-11073, Life Technologies, Eugene, OR, USA), goat anti-rabbit secondary antibody conjugated to AlexaFluor 594 (1:500, A-11012, Life Technologies), goat anti-rabbit marina blue (1:500, M10992, Molecular probe), donkey anti-rabbit conjugated to AlexaFluor 488 (1:500, A-21206, Life Technologies), goat anti-chicken conjugated to DyLight 488 (1:500, 072-03-24-06, KPL, Gaithersburg, MD, USA), or goat anti-mouse secondary antibody conjugated to AlexaFluor 594 (1:500, A-11032, Life Technologies, Carlsbad, CA, USA) for 2 h at room temperature.

Sections were then washed and mounted with Fluoromount-G (Southern Biotech, Birmingham, AL, USA). Widefield images were acquired using an Infinity camera (Lumenera Co., Ontario, ON, Canada) and epifluorescence microscope (Nikon Eclipse Ci) with the Lumenera Infinity Analyze software (Lumenera Co.). For confocal images equivalent regions, 1024 × 1024 pixels, were captured using a Leica TCS SP8 MP confocal microscope using a 20× or 40× objective at 2× zoom. The region of interest was scanned at 0.3 μm intervals along the z-axis and an optical section (3.88 μm thick) was taken from each tissue section.

Volocity image analysis software (Quorum Technologies, Lewes, UK) was used to measure surface volume for GluD1 around the PKCδ cell type, co-localization of GluD1 with vGluT1, vGluT2 and Cbln1, and the quantification of puncta number of vGluT1, vGluT2, Cbln1, and GluA1. Objects were identified within respective channel and gated/thresholded based on signal intensity and size range (≥0.1–0.3 µm^3^). Noise reduction was performed using specific medium filter. This protocol was used for puncta quantification for vGluT1, vGluT2, and Cbln1. For measuring colocalization, overlapping/coinciding objects in the same voxel were identified and reported. For GluD1-PKCδ volume analysis 3D opacity mode was used. GluD1 punctate elements were detected using the appropriate channel and thresholding. For detecting PKCδ neurons, objects less than 20 µm^3^ were excluded and fill holes in objects feature was used. Thereafter parameters of “Exclude GluD1 not touching PKCδ cell” and “PKCδ cell not touching GluD1” were used to exclusively quantify total volume of GluD1 wrapped on the cell surface (GluD1+PKCδ). GluD1 volume was calculated by subtracting PKCδ cell volume from total volume. These results were also verified by manually summating volume of GluD1 punctate elements around PKCδ cell surface. Object identification for GluA1 quantification was done in an extended focus mode. The quantification of GluA1 was done by selecting ROI for every cell and the number of GluA1 puncta/cell was measured. Images were acquired from random fields with cells that showed full PKCδ outline. For non-cell puncta analysis, images were analyzed for total puncta. The average of cells/images per animal was calculated and volume or puncta number per animal was used for further comparison between genotype or treatment. The slides were labeled with a coding system and analyzed by the experimenter blind to the genotype/treatment; groups were revealed at the end of analysis. Image depiction for representatives as well as 3D reconstruction was done using surface module of Imaris software 8.4.1, a 3D imaging software (Bitplane, South Windsor, CT, USA). The video representing the close apposition of GluD1 on PKCδ cell surface was done using animation mode of Imaris software.

### 2.3. Whole-Cell Electrophysiology

Mice: Whole-cell electrophysiology was performed as previously described [40] with minor modifications. After isoflurane anesthesia, mice were decapitated and brains were removed rapidly and placed in ice-cold artificial cerebrospinal fluid (ACSF) of the following composition (in mM): 130 NaCl, 24 NaHCO_3_, 3.5 KCl, 1.25 NaH_2_PO_4_, 0.5 CaCl_2_, 3 MgCl_2_, and 10 glucose saturated with 95% O_2_/5% CO_2_. Coronal sections of 300–350 μm thickness were prepared using vibrating microtome (Leica VT1200, Buffalo Grove, IL, USA). Whole-cell patch recordings were obtained from neurons in latero capsular region of CeA (CeLC) in voltage-clamp configuration with an Axopatch 200B (Molecular Devices, Sunnyvale, CA, USA). Glass pipette with a resistance of 4–6 MΩ were filled with an internal solution consisting of (in mM) 110 cesium gluconate, 30 CsCl, 5 HEPES, 4 NaCl, 0.5 CaCl_2_, 2 MgCl_2_, 5 BAPTA,2 Na_2_ATP, and 0.3 Na_2_GTP (pH 7.35). QX314 was added to the internal solution to block voltage-gated sodium channels. The recording ACSF contained (in mM) 1.5 CaCl_2_ and 1.5 MgCl_2_. Neurons were held at a holding potential of −70 mV for mEPSCs and 0 mV for mIPSCs. mEPSCs were recorded in the presence of 0.5 μM tetrodotoxin and 100 μM picrotoxin. mIPSCs were recorded in the presence of 0.5 μM tetrodotoxin, 10 μM CNQX, and 100 μM DL-AP5. Whole-cell recordings with a pipette access resistance less than 20 MΩ and that changed less than 20% during the duration of recording were included. Signal was filtered at 2 kHz and digitized at 10 kHz using an Axon Digidata 1440A analog-to-digital board (Molecular Devices). The mEPSC and mIPSC recordings were analyzed using Minianalysis software (Synaposoft, Atlanta, GA, USA) with an amplitude threshold set at 5 pA. Frequency and amplitude of the miniature and spontaneous currents were determined. For evoked responses, a bipolar stimulating electrode (World precision instruments, FL, USA) was placed on the fiber tract dorsomedial to the CeLC, which carries afferents from PB [11,14]. Evoked EPSCs were recorded from CeLC neurons at a holding potential of −70 mV in the presence of picrotoxin (100 µM). Thirty consecutive trials were recorded at 0.1 Hz for each condition. Amplitudes of AMPA receptor-EPSCs were calculated by averaging 30 EPSCs at each condition. For recording cell excitability, current-clamp recordings were conducted as previously described [48].

Rats: Coronal (400 μm) brain slices containing the CeA of the right hemisphere were obtained as described previously [49,50]. The right hemisphere was selected because of evidence for lateralized amygdala function in pain (see [51,52,53]). Brains were immersed in ice-cold oxygenated physiological ACSF solution (87 NaCl, 75 sucrose, 25 glucose, 5 KCl, 21 MgCl_2_, 0.5 CaCl_2_, and 1.25 NaH_2_PO_4_). Brain slices were prepared using a Vibratome (VT1200S, Leica Biosystems, Nussloch, Germany) and incubated in oxygenated ACSF at 35 °C for 20 min, and then at room temperature (21 °C) for at least 40 min. One slice was transferred to the recording chamber and superfused by ACSF (31 ± 1 °C) at ~2 mL/min. Whole-cell voltage- and current-clamp recordings were made from visually identified CeLC neurons using DIC-IR video microscopy as described previously [49,50]. Borosilicate glass electrodes with tip resistances of 6–8 MΩ were filled with a potassium gluconate based internal solution containing (in mM): 122 K-gluconate, 5 NaCl, 0.3 CaCl_2_, 2 MgCl_2_, 1 EGTA, 10 HEPES, 5 Na_2_-ATP, and 0.4 Na_3_-GTP; pH adjusted to 7.2–7.3 with KOH and osmolarity to 280 mOsm/kg with sucrose. For data acquisition and analysis, a low-noise Digidata 1322A interface (Axon Instruments, Molecular Devices, San Jose, CA, USA), a dual 4-pole Bessel filter (Warner Instruments, Hamden, CT, USA), Axoclamp-2B amplifier (Axon Instruments), and pClamp10 software (Axon Instruments) were used. If series resistance (monitored with pClamp10 software) changed >20%, the neuron was discarded. In current clamp mode, action potentials were evoked from a holding potential of −60 mV using 0.5 s depolarizing current steps of increasing amplitude. Current injections were done to set the resting potential to −60 mV for all cells. We also measured excitability at resting membrane potential without basal current injection and found similar results (data not included). Rheobase was defined as the minimal depolarizing current (0.5 s, 10 pA steps) that induces an action potential.

### 2.4. Synaptoneurosome Preparation and Western Blot Analysis

For synaptoneurosomal preparation, mice were anesthetized using isoflurane anesthesia, mice were then decapitated and thereafter all experimental procedures were conducted on ice. The right CeA was punched and put into ice cold synaptoneurosomal buffer (10 mM HEPES, 1 mM EDTA, 2 mM EGTA, 0.5 mM DTT, 10 µg/mL leupeptin, and 50 µg/mL soybean trypsin inhibitor, pH 7.0) additionally containing 5 mg/mL pepstatin, 50 mg/mL aprotonin, and 0.5 mM phenylmethanesulfonylfluoride (PMSF). The tissue was thoroughly homogenized. The homogenate was diluted further with the same volume of synaptoneurosome buffer and briefly and gently sonicated, delivering 3 pulses using an output power of 1 Sonic dismembrator Model 100 (Fisher Scientific, Waltham, MA, USA). The sample was loaded into a 1.0 mL Luer-lock syringe (BD syringes) and filtered twice through three layers of a pre-wetted 100 µm pore nylon filter CMN-0105-D (Small Parts Inc., Logansport, IN, USA) held in a 13 mm diameter filter holder XX3001200 (Millipore, MA, USA). The resulting filtrate was loaded into a 1 mL Luer-lock syringe and filtered through a pre-wetted 5 µm pore hydrophilic filter CMN-0005-D (Small Parts Inc., Logansport, IN, USA) held in a 13 mm diameter filter holder. The resulting filtrate was centrifuged at 1000× *g* for 10 min. The pellet obtained corresponded to the synaptoneurosome fraction. Isolated synaptoneurosomes were resuspended in 75 µL of buffer solution containing 0.32 M sucrose, and 1 mM NaHCO3 (pH 7.0).

For Western blotting, synaptoneurosomes were loaded on 10% Sodium dodecyl sulfate gel in equal amounts (15–30 µg/well). The samples were run at 114 volts for a duration of 1 h. Gels were transferred to nitrocellulose membrane (GE Healthcare, Piscataway, NJ, USA), a wet transfer was carried out. The voltage for transfer was kept at 114 volts and the duration for which transfer was carried out was 1 h and 15 min. Electrophoresis and transfer apparatuses used were the Biorad mini protean tetra cell (Bio-Rad Laboratories, Inc., Hercules, CA, USA). Transfer was followed by blocking with 5% milk in Tris-buffered Saline with 1% Tween 20 (TBST) for 1 h at room temperature. The primary antibodies, rabbit anti-GluD1 (AGC-038, Alomone labs), 1:1000; guinea pig anti-pan-AMPAR (Af580, Frontier Institute Co., Ltd.) were used and kept overnight for incubation at 4 °C. After primary antibody incubation, the blots were washed and incubated with horse-radish peroxidase (HRP) conjugated anti-rabbit secondary antibody, 1:5000 (Cell Signaling Technology, Danvers, MA, USA) or HRP conjugated anti-guinea pig (#AP108P, Sigma-Aldrich), 1:10,000, for 1 h at room temperature followed by washing with TBST. Blots were developed using enhanced chemiluminescent (ECL) Plus Western Blotting Detection System kit RPN2132 (GE Healthcare, Piscataway, NJ, USA). Blots were analyzed using X-ray film processor or FluorChem Q (Proteinsimple) or Chemidoc (Biorad). For analysis of protein expression, the arbitrary optical density of each sample was normalized to β-actin.

### 2.5. Stereotaxic Surgery

#### 2.5.1. Stereotaxic AAV Injection

Injection of AAV was conducted as previously described [48] with minor modifications. Briefly, mice were anesthetized with isoflurane and placed in a stereotaxic frame (51733U, Stoelting, Wood Dale, IL, USA). The skull was exposed, and a small hole was drilled through the skull at the coordinates for central amygdala (AP: −1.22 mm, ML: ±2.9 mm, DV: −4.6 mm). The designer receptors exclusively activated by designer drug (DREADD) virus particles AAV-hSyn-DIO-hM3D (Gq) or AAV-hSyn-DIO-hM4D (Gi)- mCherry (Neurophotonics, Quebec, QC, Canada) or AAV9.hsyn. eGFP/AAV9.hsyn. eGFP-Cre (University of Pennsylvania vector core) were injected by using microliter syringe (NanoFil, World Precision Instruments, Sarasota, FL, USA) with a 33-gauge beveled needle (NF33BV-2, World Precision Instruments). The injection needle was lowered, and virus particles were delivered at a rate of 1nl/sec using a UMP3 micro-syringe pump (World Precision Instruments). The volume of injections was kept at 150 nL to obtain the precise local infection and to avoid the leak into other brain regions. The needle was left in place for additional 10 min at the injection site and was slowly withdrawn over the period of 5 min. The incision was sealed with surgical tissue adhesive (1469SB, 3M, Maplewood, MN, USA). Only mice that had virus injection restricted to the CeA were included in the study.

#### 2.5.2. Cannulation

Cannulation surgery was performed as previously described [48] with minor modifications. Mice were anesthetized with isoflurane and placed in a stereotaxic frame. The skull was exposed, a small hole was drilled through the skull, and the 26-gauge stainless steel guide cannula was implanted unilaterally above the lateral ventricle for ICV administration. The stereotaxic coordinates were as follows; AP: −0.22 mm, ML: +0.8 mm, DV: −2.3 mm. For bilateral CeA cannulation, guide cannula was implanted at the stereotaxic coordinates as follows: AP: −1.22 mm, ML: ±2.75 mm, DV: −4.0 mm. The guide cannulas were secured to the skull with stainless steel screws and dental acrylic cement. The animals were allowed to recover for a period of 10 days before being used for experiments. Cannula and viral injection locations were verified after the end of behavioral experiments by examining the fixed brain tissue from these animals under light or fluorescent microscope.

### 2.6. Pain Induction Models

#### 2.6.1. CFA Inflammatory Pain Model

Inflammation was induced by intraplantar injection of 10 μL of complete Freund’s adjuvant (CFA; 0.5 mg/mL heat-killed M. tuberculosis; Sigma, St. Louis, MO, USA) into the hind paw. Saline (pH 7.4) served as control. Mechanical sensitivity was measured using electronic von Frey filament at multiple time points (6 h to 1 week) after CFA injection.

#### 2.6.2. Spinal Nerve Ligation Pain Model

The spinal nerve ligation (SNL) model of chronic neuropathic pain was used as previously described [21,54]. Surgical procedure was conducted under isoflurane anesthesia. L5 spinal nerve was isolated and tightly ligated with 6–0 silk thread. The muscles were sutured, closed, and the skin was clipped together. Antibiotic bacitracin was applied, and animals were monitored post-surgery for any signs of distress. In sham operated control animals, same surgical procedure was performed without spinal nerve ligation or irritation.

### 2.7. Drug Administration

#### 2.7.1. Recombinant Cerebellin 1 (Cbln1) Administration

In mice, recombinant Cbln1 (6934-CB-025, R&D systems, Minneapolis, MN or 00361-03-100, Aviscera Bioscience, Santa Clara, CA, USA) or Cbln2 (7044-CB-050, R&D systems) was administered into the CeA (through cannula) at a dose of 250 ng in 500 nL (or 250 nL for some experiments) per side in sterile PBS. For intracerebroventricular injection a dose of 1.5 µg in 1.5 µL was used. Changes in mechanical hypersensitivity were measured using von Frey test up to a week after Cbln1 administration. In rats with neuropathic pain, Cbln1 (500 ng in 1 µL) was administered unilaterally in right CeA (coordinates: 2.5 mm caudal to bregma, 4.0 mm lateral to midline, and 7.5 mm deep) using a 5 µL Hamilton syringe (33 gauge) while the animals were anesthetized with isoflurane (5%, induction; 2%, maintenance; precision vaporizer, Harvard Apparatus, Holliston, MA, USA) and placed in a stereotaxic frame (David Kopf Instruments, Tujunga, CA, USA). Changes in vocalization, averse and affective behaviors were recorded up to a week after drug administration. The right CeA was selected because previous reports have shown right hemisphere lateralization of amygdala function in the modulation of tactile hypersensitivity in rodents [51].

#### 2.7.2. Chemogenetics

Chemogenetic experiments were conducted as previously described [48] with minor modifications. After habituation to the room and von Frey testing apparatus, DREADD injected animals received intraplantar CFA injection for pain induction (or saline in case of control). Mechanical hypersensitivity was tested 48 h after intraplantar CFA/saline injection and thereafter animals received intraperitoneal injection of 1 mg/kg of clozapine-N-oxide in saline (CNO; Hello Bio Inc., Princeton, NJ, USA) or saline alone and mechanical hypersensitivity was measured at 1 h and 24 h.

### 2.8. Behavioral Assessment Tests in Mouse

#### 2.8.1. Von Frey Filament Test

Von Frey filament test in mice was performed using an electronic von Frey anesthesiometer (IITC systems). Animals were habituated to custom testing chambers with a perforated bottom to provide access to their paws. Next, animals were habituated to the application of rigid filament (from IITC systems) applied perpendicularly to the plantar surface of the hind paw. Paw withdrawal threshold was measured as the force at which the animal showed nocifensive responses, such as brisk paw withdrawal, licking, or shaking of the paw. An average of 3 readings were taken for each time point.

#### 2.8.2. Tail Flick Test

Tail flick test was performed using IITC tail flick analgesia meter. The mice were habituated to the instrument. During the test, mice were restrained in a custom cylindrical chamber and the middle portion of the tail was exposed to radiant beam of light that served as the heat stimulus (~50 °C). The latency for the animal to flick the tail out of the beam of light was recorded. An automatic cut-off latency of 20 s was set to prevent tissue damage.

#### 2.8.3. Hotplate Test

Hotplate test was performed using the IITC Hot plate analgesia meter. Mice were placed on the metal surface maintained at 55 °C enclosed by a plastic chamber. The response latency for paw licking or jumping behavior was recorded. The animal was removed from the hotplate after the nociceptive response or a cut-off latency of 20 s to prevent tissue damage.

#### 2.8.4. Gabapentin Induced Conditioned Place Preference

Conditional place preference (CPP) apparatus consisting of two equal sized chambers (20 cm × 20 cm × 20 cm) with distinct contextual characteristics was used for CPP study. On day 1, mice were habituated for 30 min with free access to both the chambers. On day 2, the baseline preference for each chamber was recorded for 15 min (pretest). After pretest readings were determined, gabapentin was paired with the non-preferred chamber, and saline was paired with the preferred chamber. On the conditioning day, day 3, mice received an intraperitoneal injection of saline and after 10 min were restricted in preferred chamber for 30 min in morning. At least 4 h after the saline was injected, the same mice were injected with gabapentin (100 mg/kg) and after 10 min were placed in nonpreferred chamber. On day 5, for post-test, mice were placed in the CPP box with the door open to have free access to both the chambers for 15 min and the number of entries and time spent was noted. CPP score was calculated by subtracting the time spent in gabapentin paired chamber on the pretest day from that of the posttest (CPP score = Posttest − Pretest).

The Cbln1-induced conditioned place aversion (CPA) used the same apparatus and similar day 0 and 1 procedures were carried out. Thereafter, on day 2 (conditioning day), two conditioning sessions were carried out. The control group received the vehicle (PBS 500 nL/side, intra-CeA) in both the non-preferred and preferred chamber in the morning and evening session, respectively. On the other hand, the Cbln1 treatment group received the vehicle (PBS 500 nL/side, intra-CeA) in the non-preferred chamber in the morning session and Cbln1 (250 ng/500 nL/side, intra-CeA) in the preferred chamber in the evening session. The mice were restricted to each chamber for 1 h during each session. On day 4 (After 48 h of Vehicle/Cbln1 treatment), post-test was carried out. The mice were placed in the CPA box with the door open to have free access to both the chambers for 15 min and the time spent in each chamber was recorded. Change in preference was calculated by subtracting the time spent in preferred chamber on the pretest day from that of the posttest (Change in preference = Posttest − Pretest).

#### 2.8.5. Formalin Test

The formalin test was carried out in a square plexiglass chamber; 10 μL of 5% formalin was administered into the plantar surface of the right hind paw. The control mice were injected with 10 μL of saline. Each mouse was immediately placed in the observation chamber after injection, and the time licking the injected hind paw was recorded during the acute (0 to 15 min) and delay (15 to 30 min) phases.

#### 2.8.6. Fear Conditioning

Fear conditioning was performed as previously described [46] with some modifications. For fear conditioning, mice were placed in a plexiglas rodent conditioning chamber (chamber A; model 2325-0241 San Diego Instruments, San Diego, CA, USA) with a metal grid floor, enclosed in a sound-attenuating chamber and illuminated with white light. On day 0, mice were habituated to the chamber for 30 min. On the day of conditioning (day 1), mice were placed in the chamber for 3 min followed by five CS–US pairings (5 CS-US). The CS was a tone (85 db, 3 kHz) delivered for 30 s with a 1 min inter-trial interval (ITI). The US was a 0.5 mA foot-shock delivered for 2 s that terminated together with the CS. Mice were removed from chamber 2 min after the final CS-US pairing. On testing day 2 (post-test), mice were placed in the novel Plexiglas chamber with different olfactory and visual cues and solid plexiglass floor and were exposed to the CS (tone) for 2 min. Behavioral freezing events were measured as absence of all non-respiratory movements every five seconds during the CS presentation on conditioning (day 1) and post-test (day 2) sessions. Percent freezing was calculated as the ratio of number of events spent freezing over total number of events times 100.

### 2.9. Behavioral Assessment Tests in Rats

#### 2.9.1. Vocalization

Rat vocalizations in the audible and ultrasonic (25 ± 4 kHz) ranges were measured using a condenser microphone and a bat detector, respectively, which were placed in front of the animal at a fixed distance in a custom designed recording chamber (U.S. Patent 7213538) [21,55]. Animals were anesthetized briefly with isoflurane (2%) and placed in a custom-designed recording chamber with openings for head and limbs. After recovery and habituation to the chamber, mechanical stimuli of innocuous (100 g/6 mm^2^) and noxious (500 g/6 mm^2^) intensities were applied to the hind paw (15 s) using calibrated forceps with a force transducer to monitor the applied force (in g). Durations of audible and ultrasonic vocalizations were analyzed for 1 min using Ultravox 2.0 software (Noldus Information Technology).

#### 2.9.2. Von Frey Test

The von Frey test in rats was performed with a series of von Frey filaments (Touch Test sensory evaluators; Stoelting, Wood Dale, IL, USA), and withdrawal threshold was calculated using the Dixon up–down method [56]. Hind paw withdrawal threshold was tested by perpendicular application of the filaments to the plantar surface of the left (ipsilateral to SNL) paw; the cutoff filament was 15 g.

#### 2.9.3. Elevated plus Maze

Elevated plus maze (EPM, Columbus Instruments, (arm length, 50 cm; arm width, 10 cm; and wall height, 40 cm) was used to test for anxiety-like behaviors. After habituation to the behavior room for 1 h, the animal was placed in the central area of the EPM, facing an open arm. Movement in the open and closed arms of EPM were measured as number of entries into the respective arm, for 15 min using a computerized analysis system (Multi-Varimex software; Columbus Instruments, Columbus, OH, USA). The ratio of open-arm entries to the total number of entries calculated as percentage during the first 5 min is reported [55].

### 2.10. Statistics

All data are presented as mean ± SEM. Data were analyzed using either Student’s parametric two-tailed unpaired t-test, one-way ANOVA or two-way ANOVA with post hoc multiple comparisons test. Differences were considered significant if *p* < 0.05. Prism 7 (GraphPad Software Inc., San Diego, CA, USA) was used for analysis. Sample sizes were based on our previous analysis using similar methods. Blinding was employed for immunohistochemical and behavioral tests using a coding system.

## 3. Results

### 3.1. Unique Cell- and Input-Specific Expression of GluD1 in the Central Amygdala

**Figure 1:**
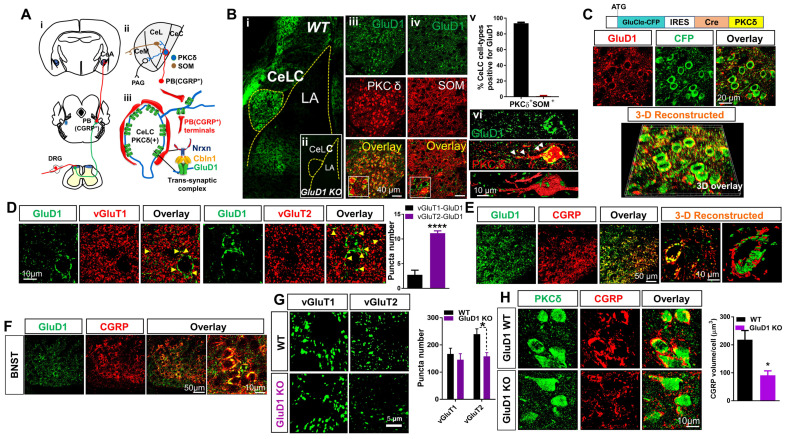
Glutamate receptor delta 1 (GluD1) serves as a critical regulator at parabrachio-amygdalar synapses. (**A**) Schematic representation of parabrachio-amygdalar pathway. (i) Nociceptive information from periphery reaches laminae I and V spinal dorsal horn neurons. Ascending projections transmit nociceptive information via lateral parabrachial nucleus (PB) to the central amygdala (CeA). (ii and iii) Lateral and capsular divisions of the CeA (CeLC) receive glutamatergic inputs from PB-CGRP^+^ terminals. GluD1 is localized postsynaptically and Neurexin 1 (Nrxn) is the presynaptic partner of the intermediate protein Cbln1. (**B**) (i) Immunohistochemical analysis reveals enriched expression of GluD1 in CeLC. (ii) No labeling in GluD1 KO demonstrates antibody specificity. (iii) GluD1 shows perisomatic and punctate labeling on PKCδ^+^ neurons. (iv) Negligible co-labeling with SOM^+^ neurons. (v) Quantification of GluD1 colocalization in both neuronal populations reveals 93.49% ± 1.104% were PKCδ^+^, whereas only 0.788% ± 0.572% were SOM^+^ (*n* = 5 mice). (vi) The 3D reconstruction of GluD1 puncta on PKCδ^+^ neurons. (**C**) GluD1 localization to PKCδ^+^ neurons using the PKCδ-CFP-Cre reporter mouse line. (**D**) Higher colocalization (yellow arrows) of GluD1 with PB marker vGluT2 vs. BLA marker vGluT1 (vGluT1-GluD1 (*n* = 5 mice): 2.74 ± 0.93; vGluT2-GluD1 (*n* = 5 mice): 11.16 ± 0.48, **** *p* < 0.0001, two-tailed unpaired t-test). (**E**) GluD1 is postsynaptic to CGRP terminals. Confocal analysis and 3D reconstruction reveal close apposition of GluD1 with CGRP. (**F**) GluD1 and CGRP show colocalization in BNST. Immunohistochemistry for GluD1 and CGRP in BNST demonstrate that CGRP ensheathes perisomatic GluD1 with a basket-like structure, similar to the pattern observed in CeLC. (**G**) Selective reduction in vGluT2 puncta in CeLC in GluD1 KO mice (Two-way ANOVA, genotype F (1, 12) = 4.774, *p* = 0.022, vGluT1: WT vs. GluD1 KO: 166.750 ± 21.379 vs. 145.5 ± 22.29; vGluT2: WT vs. GluD1 KO: 239 ± 19.759 vs. 158.25 ± 13.665, Bonferroni’s multiple comparisons test, * *p* = 0.025, *n* = 4 mice per group). (**H**) GluD1 deletion leads to significant reduction in CGRP projection in CeLC. Immunohistochemistry for CGRP in WT and GluD1 KO exhibit significant reduction in CGRP in CeLC, suggesting an impairment in PB inputs. (WT (*n* = 5 mice) vs. GluD1 KO (*n* = 4 mice): 218.4 ± 33.0 vs. 91.1 ± 15.9, * *p* = 0.015, two-tailed unpaired *t*-test).

We examined the expression of GluD1 in the CeA along the spino-parabrachio-amygdala pathway (Figure 1A), which comprises of glutamatergic PB neurons including CGRP+ cells projecting to CeLC. Strong expression of GluD1 was observed in CeA. The expression was found to be both punctate as well as perisomatic (Figure 1Bi, vi) and observed in CeC and CeL regions but not in CeM region. We next examined the cell type–specific expression of GluD1. Two predominant non-overlapping cell types in the CeA include those expressing PKCδ or SOM. Majority of perisomatic and punctate GluD1 elements were found to localize with PKCδ^+^ neurons, as also evident from the 3D-reconstructed images (Figure 1Biii–vi, Figure S1A video). GluD1 did not localize to either the soma or dendrites of SOM neurons. Localization of GluD1 on PKCδ^+^ neurons was confirmed using a PKCδ reporter model (Figure 1C). This reporter model expresses GluCl-CFP under the PKCδ promoter. Labeling for CFP together with GluD1 revealed co-localization, further demonstrating localization of GluD1 with PKCδ^+^ neurons.

We next examined whether GluD1 is localized to specific projections to CeA, which may further suggest its functioning as a synaptic organizer. CeA receives major excitatory projections from PB and basolateral amygdala (BLA), which can be distinguished by their expression of vGluT2 and vGluT1, respectively [57]. GluD1 was found to primarily localize with PB projections as evidenced by stronger vGluT2 co-localization compared to BLA projections which are vGluT1^+^ (Figure 1D). Although comparatively lower, the vGluT1^+^ localization of GluD1 may indicate expression at BLA or cortical or other inputs. Evidence suggests that PKCδ^+^ neurons are the main targets of synapses from PB-CGRP-positive neurons [23,24,25,26,27,58], while SOM+ neurons primarily receive inputs from PB-CGRP-negative neurons [28,29,30]. We found GluD1 elements on the cell soma in close apposition with CGRP elements and 3D reconstruction suggested the localization of CGRP to be presynaptic (Figure 1E). A similar pattern was observed in the extended amygdala bed nucleus of the stria terminalis (BNST) region (Figure 1F). Together these results suggest that in the CeA, GluD1 is primarily expressed postsynaptically at PB-PKCδ^+^ synapses.

### 3.2. GluD1 Is Necessary for Normal Excitatory Neurotransmission at the Parabrachio-Amygdala Synapses

**Figure 2:**
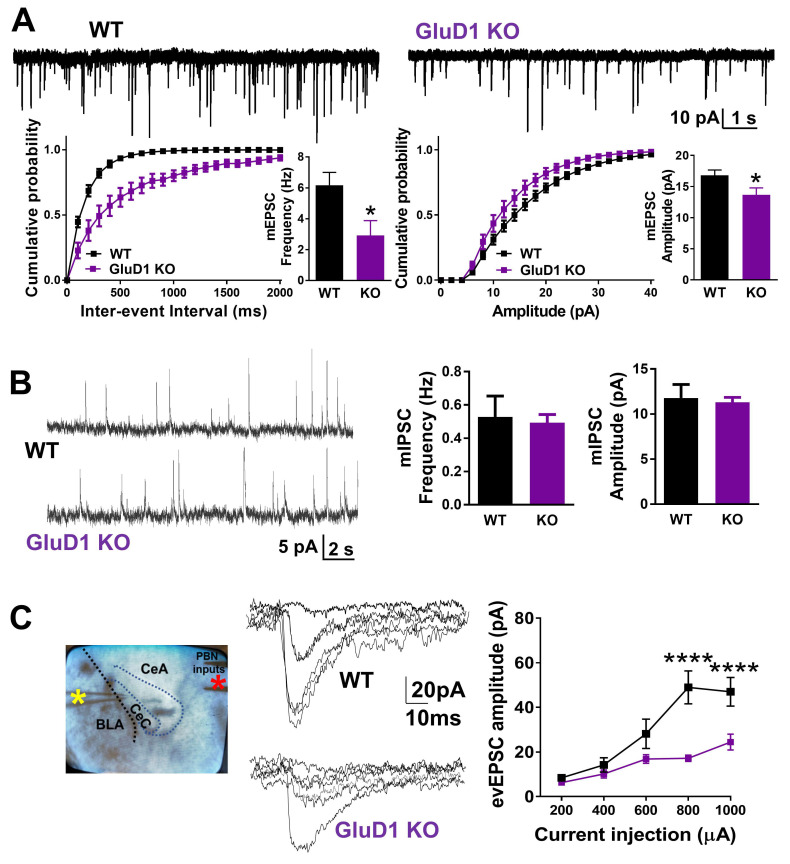
GluD1 is critical for regulating excitatory neurotransmission at parabrachio-amygdala synapses. (**A**) Whole-cell mEPSC recordings at −70mV (in the presence of picrotoxin and tetrodotoxin) from CeC neurons demonstrate significantly lower mEPSC frequency and amplitude in GluD1 KO (WT (*n* = 8) vs. GluD1 KO (*n* = 9): Frequency: 6.17 ± 0.83 Hz vs. 2.91 ± 0.96 Hz, * *p* = 0.022, two-tailed unpaired t-test with Welch’s correction; Amplitude: 16.78 ± 0.869 pA vs. 13.66 ± 1.124 pA, * *p* = 0.045, unpaired *t*-test). (**B**) No change in mIPSC frequency and amplitude in CeC due to deletion of GluD1 (Frequency: *p* = 0.74, Amplitude: *p* = 0.77; two-tailed unpaired t-test, *n* = 8). (**C**) Whole-cell evoked EPSCs from CeC neurons at -70 mV in the presence of picrotoxin with recording electrode in CeC (yellow asterisk) and stimulating electrode on PB fibers (red asterisk). The input–output curve shows significant reduction in evoked EPSC amplitude in GluD1 KO mice. (Two-way repeated measures ANOVA, genotype F (1, 28) = 23.03, *p* < 0.0001; Bonferroni’s multiple comparisons test: WT (*n* = 15) vs. GluD1 KO (*n* = 25); 800 µA current injection: 48.97 ± 7.39 vs. 17.16 ± 1.43, **** *p* < 0.0001; 1 mA current injection: 47.00 ± 6.40 vs. 24.46 ± 3.56, **** *p* < 0.0001).

We next examined whether GluD1 is obligatory for normal excitatory function in the CeA. We first tested whether there were changes in excitatory terminals in the CeA of GluD1 KO mice. A reduction in vGluT2 puncta and CGRP^+^ terminals but not vGluT1 puncta was found in GluD1 KO suggesting reduced PB- but not BLA-projection synapses (Figure 1G,H). Next, we examined if there were any changes in excitatory neurotransmission in CeA in GluD1 KO mice. GluD1 deletion led to a reduction in mEPSC frequency as well as amplitude in CeLC neurons (Figure 2A). This effect was more robust in the CeC compared to CeL (Supplementary Figure S2A), which corresponds to the distribution of CGRP^+^ projections. No change in inhibitory neurotransmission (Figure 2B) or excitability (Supplementary Figure S2B) of CeC neurons was observed in GluD1 KO mice. Excitatory neurotransmission at the PB-CeLC synapses was also reduced in GluD1 KO condition when evoked responses were studied (Figure 2C). These results suggest an important role of GluD1 for structural and functional integrity of the PB-CeLC synapse.

### 3.3. Downregulation of GluD1-Cbln1 in the CeLC in Inflammatory and Neuropathic Pain Models

**Figure 3:**
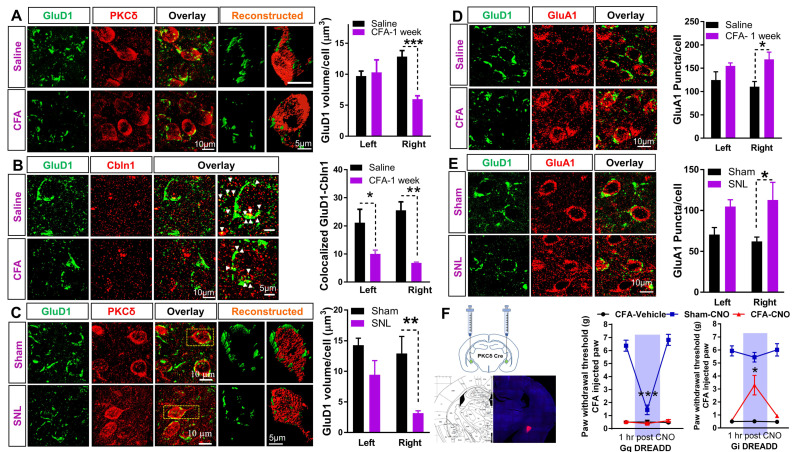
Inflammatory pain induces changes in GluD1, Cbln1, and GluA1 behavior. (**A**) GluD1 and PKCδ staining in CeLC was performed at 1 week after intraplantar CFA administration. Three-dimensional reconstruction and volume analysis of GluD1 elements in apposition with PKCδ^+^ soma was performed. Significant reduction in somatic GluD1 volume specifically in the right CeA in CFA mice (Two-way ANOVA, treatment x side interaction F (1, 17) = 10.03, *p* = 0.0056, treatment F (1, 17) = 7.08, *p* = 0.016, Saline (*n* = 4 mice left and 8 mice right) vs. CFA (*n* = 4 mice left and 5 mice right): left CeA: 9.71 ± 0.81 vs. 10.30 ± 2.01; right CeA: 12.85 ± 0.97 vs. 5.97 ± 0.56, Bonferroni’s post hoc test, *** *p* = 0.0005). (**B**) GluD1 and Cbln1 co-labeling in CeLC performed 1 week after intraplantar CFA administration. A substantial reduction in GluD1-Cbln1 colocalized puncta number in right CeA is observed (Two-way ANOVA, treatment F (1, 12) = 26.65, *p* = 0.0002, Right CeA: Saline (*n* = 4 mice): 25.5 ± 3.06 vs. CFA (*n* = 4 mice): 6.83 ± 0.24, * *p* = 0.024, Bonferroni’s post hoc test). Reduction in GluD1-Cbln1 colocalized puncta is also observed on left CeA (*p* = 0.037). (**C**) GluD1 and PKCδ co-labeling in CeLC performed 4 weeks after sham or SNL surgery in rats along with 3D reconstruction. Significant reduction is observed in perisomatic GluD1 volume in right CeA (Two-way ANOVA, treatment F (1, 12) = 14.66, *p* = 0.0024, Right CeA: Sham (*n* = 4 rat): 12.92 ± 2.77 vs. SNL (*n* = 4 rats): 3.17 ± 0.37, ** *p* = 0.007, Bonferroni’s post hoc test). No significant change in left CeA GluD1 volume (*p* = 0.19). (**D**) GluD1 and AMPA receptor subunit GluA1 co-labeling in CeLC performed 1 week after intraplantar CFA administration. An upregulation of surface GluA1 is observed after CFA treatment (Two-way ANOVA, treatment F (1, 12) = 11.06, *p* = 0.0060, Saline (*n* = 4 mice) vs. CFA (*n* = 4 mice): Right CeA: 110.48 ± 11.00 vs. 168.98 ± 15.44, * *p* = 0.018 Bonferroni’s post hoc test. No significant difference in left CeA (*p* = 0.26). (**E**) Upregulation of GluA1 subunit in CeLC of SNL mice (Two-way ANOVA treatment F (1, 12) = 11.37, *p* = 0.005, Sham (*n* = 4 rats) vs. SNL (*n* = 4 rats): Right CeA: 62.25 ± 5.32 vs. 112.89 ± 21.47, * *p =* 0.029, with Bonferroni’s multiple comparison’s post hoc test). (**F**) Chemogenetic modulation of PKCδ neurons using PKCδ-Cre mouse line. Representative image for site verification of DREADD injection. Activation of PKCδ^+^ neurons in G_q_DREADD injected normal animals using CNO (Sham-CNO) led to an increase in mechanical sensitivity (One-way repeated measures ANOVA, treatment F (1.50, 7.53) = 64.01, *p* < 0.0001; Sham-CNO (*n* = 6 mice); pre-CNO vs. 1 h post-CNO 6.373 ± 0.428 vs. 1.441 ± 0.365, *** *p* < 0.0001, Tukey’s post hoc test). No change was observed across different time points in the other two groups (*n* = 4 mice for CFA-vehicle and 4 mice for CFA-CNO). In contrast, in CFA injected mice, inhibition of PKCδ+ neurons in G_i_DREADD injected animals using CNO (CFA-CNO) significantly rescued mechanical hypersensitivity (One-way repeated measures ANOVA, treatment F (1.03, 4.13) = 12.88, *p* = 0.0215; CFA-CNO (*n* = 5 mice); pre-CNO vs. 1 hr post-CNO: 0.591 ± 0.040 vs. 3.289 ± 0.748, * *p* = 0.0464, Tukey’s post hoc test). No difference was observed across different time points in the other groups (*n* = 6 mice for CFA-vehicle and 4 mice for Vehicle-CNO).

We examined the contribution of transsynaptic GluD1-Cbln1 signaling in inflammatory and neuropathic pain using CFA model in mice and SNL model in rats, respectively. GluD1 volume in cell soma on PKCδ^+^ neurons was measured along with punctate GluD1. Perisomatic and punctate expression of GluD1 was reduced in the inflammatory pain model (Figure 3A). Interestingly, GluD1 downregulation was observed in the right CeLC but not in the left CeLC consistent with the known lateralization of pain neuroplasticity to the right amygdala [51]. Expression levels of GluD1 binding partner Cbln1 were also reduced in the inflammatory pain model, and the change was more pronounced in the right CeA but was also observed in the left CeA (Figure 3B). Downregulation of perisomatic GluD1 volume around cell soma of PKCδ^+^ neurons was also observed in the neuropathic pain model and was more prominent in the right CeA (Figure 3C). We examined changes in the distribution of AMPA receptor subunit GluA1 in the pain models as a potential consequence of changes in GluD1. GluD1 downregulation was associated with an upregulation of AMPA receptor GluA1 subunit in the CFA and SNL models (Figure 3D,E). To link changes in GluD1 to pain behaviors, we used a chemogenetic approach. Because GluD1 is localized to PKCδ^+^ neurons, we selectively modulated the function of PKCδ^+^ neurons to determine its effect on inflammatory pain behaviors. CNO-induced activation of PKCδ^+^ neurons expressing Gq DREADD in normal mice that received intraplantar saline injections induced mechanical hypersensitivity (“Sham-CNO”, Figure 3F left). Inhibition of PKCδ^+^ neurons with Gi DREADD activation did not affect mechanical thresholds in normal mice but reduced mechanical hypersensitivity in the inflammatory pain model (Figure 3F right), confirming observations by the Carrasquillo group [29] that overactivation of PKCδ^+^ contributes to inflammatory pain behaviors. We also examined the time course of changes in GluD1 and Cbln1 expression to evaluate if they occur at the same time or if one precedes the other significantly. Examination of time-dependent changes in the GluD1 and Cbln1 expression in the right CeA in the CFA model revealed that downregulation begins as early as 6 h and persists for at least 1 week (Supplementary Figure S3A,B). From the time points examined, a clear sequence of downregulation of the two proteins could not be predicted. Downregulation of GluD1 and upregulation of AMPA receptors was also confirmed in synaptoneurosomal preparations from right CeA (Supplementary Figure S3C,D).

### 3.4. Recombinant Cbln1 Rescues Mechanical Hypersensitivity in an Inflammatory Pain Model with a Unique Time Course

**Figure 4:**
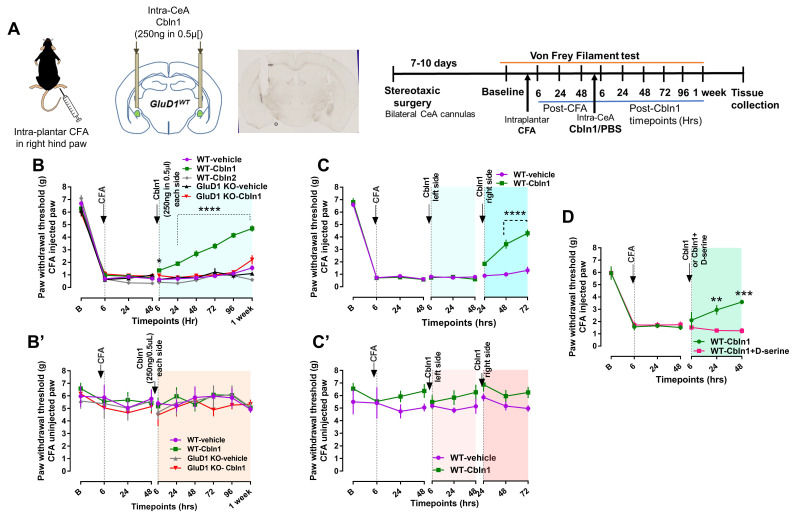
Recombinant Cbln1 administration into the central amygdala rescues behavioral hypersensitivity in an inflammatory pain model in a GluD1-dependent manner. (**A**) Experimental interventions. Mice underwent surgery for intra-CeA or intraventricular cannula implantation. After recovery, mechanical sensitivity was tested using von Frey test for paw withdrawal threshold under baseline condition followed by intraplantar injection of CFA. Effect of intra-CeA administration of Cbln1 (250 ng in 0.5 µL per side) was assessed. Paw withdrawal threshold following Cbln1 injection was measured at 6, 24, 48, 72, 96 h, and 1-week timepoints. (**B**) Alleviation of pain sensitivity after bilateral intra-CeA Cbln1 administration. Paw withdrawal threshold increased after a single Cbln1 injection and lasted up to a week; Two-way repeated measures ANOVA, treatment F (4, 22) = 68.54, *p* < 0.0001, Bonferroni’s post hoc test, WT-vehicle (*n* = 6 mice) vs. WT-Cbln1 (*n* = 7 mice); 6 h, * *p* = 0.012, 24 h–1 week, **** *p* < 0.0001. This improvement was not observed in GluD1 KO (GluD1 KO-vehicle (*n* = 4 mice), GluD1 KO-Cbln1 (*n* = 6 mice)). Cbln2 was not able to reverse the CFA-induced mechanical hypersensitivity (*n* = 4 mice). (**B’**) No change in mechanical threshold in von Frey analysis in control (non-CFA injected) paw. (**C**) Rescue of inflammatory pain by Cbln1 injection is lateralized. Recombinant Cbln1 first injected into the left CeA did not show any rescue in mechanical hypersensitivity. However, injection into the right CeA was able to rescue mechanical hypersensitivity; Two-way repeated measures ANOVA, treatment F (1, 9) = 44.15, *p* < 0.0001; Bonferroni’s post hoc test WT CFA-vehicle (*n* = 4 mice) vs. WT CFA-Cbln1 (*n* = 7 mice); 48 h and 72 h **** *p* < 0.0001. (**C’**) No change in mechanical threshold in von Frey analysis in control (non-CFA injected) paw. (**D**) D-serine (30 µg in 0.5 µL) opposes the antihyperalgesic effect of recombinant Cbln1 (250 ng in 0.5 µL). Two-way repeated measures ANOVA, treatment F (1, 6) = 5.35, *p* = 0.060, Bonferroni’s post hoc test WT-Cbln1 (*n* = 4 mice) vs. WT-Cbln1+D-serine (*n* = 4 mice); 24 h, ** *p* = 0.008; 48 h, *** *p* = 0.0001.

We next tested whether restoring the GluD1-Cbln1 signaling using recombinant Cbln1 could mitigate chronic pain. Using stereotaxic surgery, mice were bilaterally cannulated into the CeA and after a recovery of 10 days, effect of recombinant Cbln1 (250 ng in 500 nL each side) was examined in the CFA inflammatory pain model (Figure 4A). The use of 500 nL volume was based on our previous studies [59,60]. Bilateral injection of recombinant Cbln1 into the CeA mitigated mechanical hypersensitivity induced by CFA (Figure 4B). The antihyperalgesic effect gradually increased over the course of one week. Cbln1 was ineffective in mitigating hypersensitivity in GluD1 KO demonstrating the requirement for GluD1-Cbln1 signaling (Figure 4B). Cbln2 is another member of the cerebellin family of proteins with synaptogenic activity [61]. Intra-CeA Cbln2 administration was ineffective in producing antihyperalgesic effects demonstrating selectivity and a unique role of Cbln1 versus Cbln2 in the modulation of PB-CeLC synapses (Figure 4B). Pain-related hemispheric lateralization is a well-documented observation with the right CeA playing a pain facilitatory role [51,52,53] but the underlying mechanisms are poorly understood. We therefore examined whether rescue by recombinant Cbln1 shows lateralization. Injection of Cbln1 into the right, but not the left; CeA was sufficient to mitigate mechanical hypersensitivity in the inflammatory pain model (Figure 4C). Mechanical hypersensitivity was absent in the paw that was not injected with CFA (Figure 4B’,C’).

To further address the potential spread of Cbln1 in other regions and regional specificity of Cbln1 effect, we conducted control experiments. In the first set of experiments recombinant Cbln1 (250 ng) was injected at a lower volume, 250 nL on each side. In these animals, Cbln1 was able to effectively reduce mechanical hypersensitivity induced by CFA (Supplementary Figure S4A). Next, we conducted off-site control (dorsal striatum) experiments. Injection of recombinant Cbln1 into the dorsal striatum (250 ng in 250 nL on each side) did not produce a persistent rescue of mechanical hypersensitivity in the CFA injected mice (Supplementary Figure S4A). Together these sets of experiments further establish the effectiveness and regional specificity of action of recombinant Cbln1 in rescuing averse behavior in the inflammatory pain model. To further examine the selective contribution of GluD1-Cbln1 signaling in the CeA to the antihyperalgesic effect, we tested a GluD1 ligand (D-serine) in the CFA model. We hypothesized that D-serine, which binds to the ligand binding domain of GluDs and induces conformational changes [32,33], may affect GluD1-Cbln1 interaction and modulate the antihyperalgesic effect of recombinant Cbln1. When administered together with Cbln1, we found that D-serine prevented the reduction in mechanical hypersensitivity by recombinant Cbln1 (Figure 4D). These results suggest that D-serine-induced conformational changes may oppose the association of Cbln1 with GluD1 and downstream effects.

### 3.5. Recombinant Cbln1 Normalizes Synapse Structure in the CeLC in an Inflammatory Pain Model

**Figure 5:**
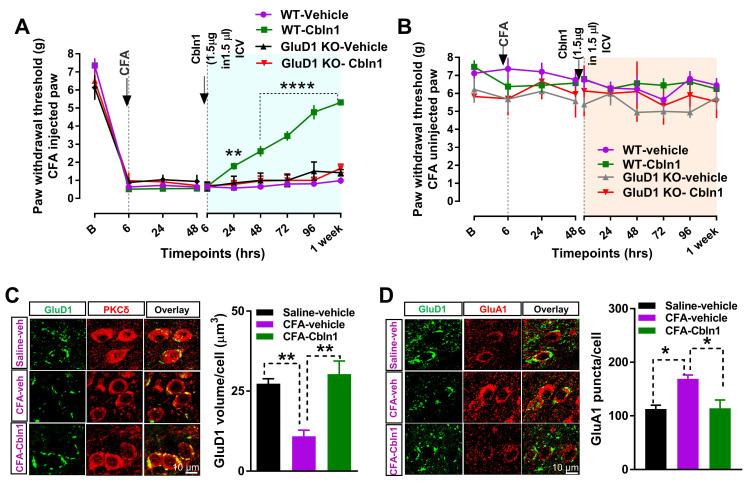
Recombinant Cbln1 administration normalizes synaptic dysfunction in the CeA in inflammatory pain. (**A**) Intracerebroventricular administration of Cbln1 (1.5 µg in 1.5 µL PBS) 48 h after CFA also attenuated mechanical hypersensitivity for at least one week. Two-way repeated measures ANOVA, F (3, 14) = 12.81, *p* = 0.0001, Bonferroni’s post hoc test, WT CFA-vehicle (*n* = 4 mice) vs. WT CFA-Cbln1 (*n* = 6 mice), ** *p* = 0.0051, **** *p* < 0.0001. No effect of Cbln1 was observed in GluD1 KO (*n* = 4 mice (vehicle), 4 mice (Cbln1)). (**B**) No change in mechanical threshold in von Frey analysis in control (non-CFA injected) paw. (**C**) Immunohistochemical analysis of right CeLC for the effect of ICV administration of recombinant Cbln1 on perisomatic GluD1 volume in the CFA pain model. Recombinant Cbln1 restored GluD1 levels in CFA mice compared to mice injected with PBS (One-way ANOVA, treatment F (2, 9) = 14.38, *p* = 0.009; Bonferroni’s multiple comparison; Saline-vehicle (*n* = 4 mice): 27.3 ± 1.53, vs. CFA-vehicle (*n* = 4 mice): 10.87 ± 1.94, ** *p* = 0.068; CFA-vehicle vs. CFA-Cbln1 (*n* = 4 mice): 30.3 ± 4.08, ** *p =* 0.0023). (**D**) Recombinant Cbln1 normalized surface GluA1 upregulation in the right CeLC in CFA pain model (One-way ANOVA treatment F (2, 9) = 9.23 *p* = 0.006; Bonferroni’s multiple comparison; Saline-vehicle (*n* = 4 mice) vs. CFA-vehicle (*n* = 4 mice): 112.9 ± 7.07 vs. 169.2 ± 6.83, * *p* = 0.013 and vs. CFA-Cbln1 (*n* = 4 mice): 114.2 ± 15.4, * *p* = 0.015).

We next examined the effect of intracerebroventricular (ICV) injection of Cbln1 on mechanical hypersensitivity in the inflammatory pain model since this may be relevant to clinical application as protein therapeutics. ICV injection of Cbln1 mitigated pain behavior in wildtype but not in GluD1 KO with a similar temporal profile as intra-CeA administration of Cbln1, pointing to the CeA as a major site of action (Figure 5A). We also examined the effect of Cbln1 in a chemotherapy-induced neuropathic pain model. Cisplatin (5 mg/kg) was injected once a week and the effect of ICV injection of Cbln1 (1.5 or 3 µg) was examined. Reduced mechanical hypersensitivity was found by Cbln1 injection (Supplementary Figure S4B), suggesting antihyperalgesic effects of Cbln1 in other pain models. Next, we examined whether recombinant Cbln1 normalized synaptic dysregulation in the CeA. We found that recombinant Cbln1 was able to restore the downregulated GluD1 in the CeLC in the CFA model to levels similar to normal animals (Figure 5C). Cbln1 also normalized the upregulated GluA1 in the CeLC in the pain model (Figure 5D). These results demonstrate that recombinant Cbln1 is able to restore normal synaptic composition in the CeA in inflammatory pain conditions.

### 3.6. Recombinant Cbln1 Mitigates Averse and Affective Behaviors in a Neuropathic Pain Model

**Figure 6:**
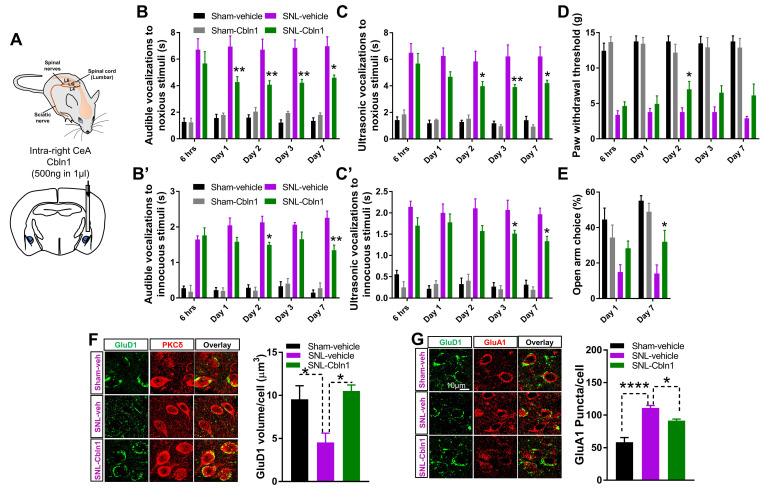
Recombinant Cbln1 rescues hypersensitivity and averse-affective behaviors in a neuropathic pain model. (**A**) Schematic representation of SNL model. The left L5 spinal nerve was ligated. Cbln1 (500 ng in 1 µL) or PBS was injected into right CeA of SNL rat. (**B**) Intra-CeA Cbln1 showed reduction in noxious stimuli induced audible vocalization in SNL rats; *n* = Sham-vehicle (6 mice), Sham-Cbln1 (5 mice), SNL-vehicle (7 mice), SNL-Cbln1 (7 mice) (Two-way ANOVA treatment F (3, 103) = 120, *p* < 0.0001; Bonferroni’s multiple comparison test, SNL-vehicle vs. SNL-Cbln1: Day 1: 6.948 ± 0.793 vs. 4.273 ± 0.410, ** *p* = 0.0012; Day 2: 6.711 ± 0.796 vs. 4.076 ± 0.299, ** *p* = 0.0015; Day 3: 6.853 ± 0.606 vs. 4.226 ± 0.251, ** *p* = 0.0016; Day 7: 6.973 ± 0.712 vs. 4.604 ± 0.202, * *p* = 0.0126). (**B’**) Injection of recombinant Cbln1 into the right CeA reduced audible vocalization to innocuous stimuli in SNL rats (Two-way ANOVA treatment F (3, 103) = 204.5, *p* < 0.0001; Bonferroni’s multiple comparison test, SNL-vehicle vs. SNL-Cbln1: Day 2: 2.130 ± 0.171 vs. 1.497 ± 0.068, * *p* = 0.0284; Day 7: 2.258 ± 0.191 vs. 1.343 ± 0.145, ** *p* = 0.0015; *n* = 5–7 rats per group). (**C**) Intra-CeA Cbln1 reduced ultrasonic vocalization in SNL rats (Two-way ANOVA treatment F (3, 103) = 119.3, *p* < 0.0001; Bonferroni’s multiple comparisons test, SNL-vehicle vs. SNL-Cbln1: Day 2: 5.845 ± 0.765 vs. 3.974 ± 0.351, * *p* = 0.0323; Day 3: 6.213 ± 0.868 vs. 3.907 ± 0.199, ** *p* = 0.0041; Day 7: 6.221 ± 0.709 vs. 4.211 ± 0.212, * *p* = 0.0340; *n* = 5–7 rats per group). (**C’**) Ultrasonic vocalization to innocuous stimuli (Two-way ANOVA treatment F (3, 103) = 172.1, *p* < 0.0001; Bonferroni’s multiple comparison test, SNL-vehicle vs. SNL-Cbln1: Day 3: 2.070 ± 0.226 vs. 1.515 ± 0.073, * *p* = 0.0408; Day 7: 1.968 ± 0.143 vs. 1.333 ± 0.115, * *p* = 0.0256; *n* = 5–7 rats per group). (**D**) Reduction in mechanical thresholds in the von Frey test were mitigated by intra-CeA Cbln1 (Two-way ANOVA treatment F (3, 103) = 147.1, *p* < 0.0001; Bonferroni’s multiple comparison test, SNL-vehicle vs. SNL-Cbln1: Day 2: 3.781 ± 0.615 vs. 7.001 ± 1.111, * *p* = 0.0498; *n* = 5–7 rats per group). (**E**) Open arm entries in the elevated plus maze test were also increased by Cbln1 in SNL rats suggesting anxiolytic effect in neuropathic pain (Two-way ANOVA treatment F (3, 40) = 18.36, *p* < 0.0001; Bonferroni’s multiple comparison test, SNL-vehicle vs. SNL-Cbln1: Day 7: 14.078 ± 4.842 vs. 31.997 ± 6.443, * *p* = 0.0399; *n* = 5–7 rats per group). (**F**) Recombinant Cbln1 restored downregulated GluD1 in CeLC in SNL rats. Immunohistochemical analysis of perisomatic GluD1 levels in right CeA was performed in tissue from SNL rats injected with PBS or recombinant Cbln1. Recombinant Cbln1 restored GluD1 levels in SNL rats to the levels of sham rats. (One-way ANOVA F (2, 9) = 7.49, *p* = 0.012; Tukey’s post hoc test, Sham-vehicle (*n* = 4 rats): 9.54 ± 1.57 vs. SNL-vehicle (*n* = 4 rats): 4.53 ± 1.08, * *p* = 0.035; SNL-Vehicle vs. SNL-Cbln1 (*n* = 4 rats): 10.51 ± 0.69; * *p =* 0.014). (**G**) Recombinant Cbln1 normalized surface GluA1 levels in SNL rats. Immunohistochemical analysis was performed in SNL rats injected with either PBS or recombinant Cbln1 for AMPA levels. Cbln1 injection reduced the upregulated GluA1 expression in SNL rats. SNL (One-way ANOVA F (2, 8) = 27.17, *p* = 0.0003; Sham-vehicle (*n* = 4 rats): 58.35 ± 7.51 vs. SNL-vehicle (*n* = 4 rats): 110.92 ± 3.70, **** *p <* 0.0001; SNL-vehicle vs. SNL-Cbln1 (*n* = 4 rats): 91.48 ± 2.40, * *p* = 0.049).

**Figure 7:**
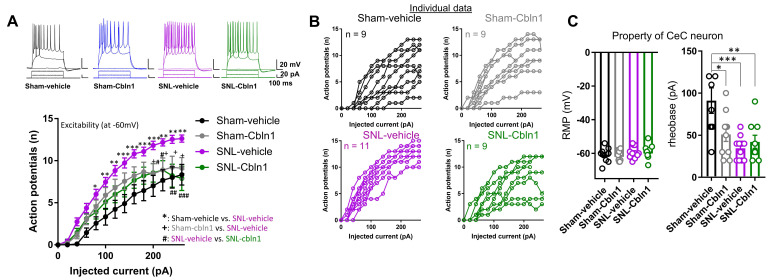
Recombinant Cbln1 rescues hyperexcitability of CeC neurons in the neuropathic pain model. (**A**) Recombinant Cbln1 reduced hyperexcitability (F-I relation) in CeC neurons in brain slices from SNL rats. Current clamp recording from CeC neurons with membrane potential set at -60 mV. Current injections were performed to generate action potentials. SNL increased the excitability and Cbln1 was able to rescue hyperexcitability in SNL model (Two-way repeated measures ANOVA, treatment F (3, 34) = 5.6, *p* = 0.003, Tukey’s multiple comparison, SNL-vehicle (*n* = 11 neurons from 5 rats) vs. Sham-vehicle (*n* = 9 neurons from 4 rats): 80 pA *p* = 0.026, 100 pA *p* = 0.0045, 120 pA *p* = 0.0011, 140 pA *p* = 0.0005, 160 pA *p* = 0.0005, 180 pA *p* = 0.0009, 200 pA *p* = 0.0005, 220 pA *p* = 0.0014, 240 pA *p* = 0.0013, 260 pA *p* = 0.0027; SNL-vehicle vs. SNL-Cbln1 (*n* = 9 neurons from 5 rats): 200 pA *p* = 0.039, 220 pA *p* = 0.037, 240 pA *p* = 0.0025, 260 pA *p* = 0.0007; Sham-Cbln1 (*n* = 9 neurons from 5 rats) vs. SNL-vehicle: 200 pA *p* = 0.031, 220 pA *p* = 0.037, 240 pA *p* = 0.034, 260 pA *p* = 0.021). (**B**) Excitability of individual cells in various Sham and SNL, control and Cbln1 groups. Note the reduced variance of individual cell excitability in SNL rats compared to other groups. (**C**) Property of CeC neurons under different conditions. A change in rheobase was observed by Cbln1 administration in Sham animals as well as due to SNL. One-way ANOVA F (3, 35) = 7.14, *p* = 0.0007; Tukey’s multiple comparison, Sham-vehicle (*n* = 9 neurons from 4 rats) vs. Sham-Cbln1 (*n* = 10 neurons from 5 rats), * *p* = 0.0231, Sham-vehicle vs. SNL-vehicle (*n* = 11 neurons from 5 rats), *** *p* = 0.0006, Sham-vehicle vs. SNL-Cbln1 (*n* = 9 neurons from 5 rats), ** *p* = 0.0053.

We further examined the effect of recombinant Cbln1 in the SNL model of chronic neuropathic pain. Rats with SNL surgery received either Cbln1 or PBS in the right CeA, 4 weeks after SNL induction, and sensory and averse-affective behaviors were measured (Figure 6A). Recombinant Cbln1 partially restored the increased audible and ultrasonic vocalizations to noxious as well as (normally) innocuous stimuli in the SNL model compared to sham (Figure 6B,B’,C,C’). Cbln1 reduced mechanical hypersensitivity in the Von Frey test in the SNL model (Figure 6D). Cbln1 had anxiolytic-like effects in the elevated plus maze test in SNL rats (Figure 6E). Thus, Cbln1 mitigates both sensory and averse-affective behaviors in a neuropathic pain condition. Furthermore, recombinant Cbln1 “normalized” both the downregulated GluD1 and the upregulated GluA1 subunit in the CeLC in the SNL model (Figure 6F,G). We further examined changes in excitability of CeLC neurons in the SNL model and effect of Cbln1. Consistent with our previous observations [50], whole-cell current clamp recordings in brain slices demonstrated that SNL led to an increase in excitability of CeLC neurons (Figure 7A), and intra-CeA administration of recombinant Cbln1 was able to reduce the hyperexcitability of CeLC neurons (Figure 7A,B). No change in resting membrane potential was observed among the different groups (Figure 7C). Rheobase current was significantly lower in the SNL and Sham-Cbln1 groups, suggesting changes in excitability (Figure 7C).

### 3.7. Cbln1 Release in the Central Amygdala in Normal Animals may Serve as a Nociceptive Signal

If downregulation of GluD1 in the inflammatory and neuropathic pain models underlies pain behaviors, genetic GluD1 ablation may result in changes in pain sensitivity in the absence of a pain condition caused by tissue pathology. To test this hypothesis, wildtype and GluD1 KO mice were characterized for pain behaviors. No change in thermal sensitivity (tail flick test, hot plate test), mechanical sensitivity (von Frey test) or conditioned place preference to gabapentin was observed in GluD1 KO (Supplementary Figure S5A–D). We found a modest increase in nocifensive behavior in the formalin pain test in GluD1 KO (Supplementary Figure S5E). The change in GluD1 KO was not observed when GluD1 KO mice were tested in the persistent CFA pain model (Figure 4B and Figure 5A). Together these data suggest that GluD1 ablation or downregulation in normal animals, i.e., in the absence of enhanced synaptic input, is not sufficient to replicate the full spectrum of changes in pain conditions but may increase susceptibility to sub-acute stimuli.

**Figure 8:**
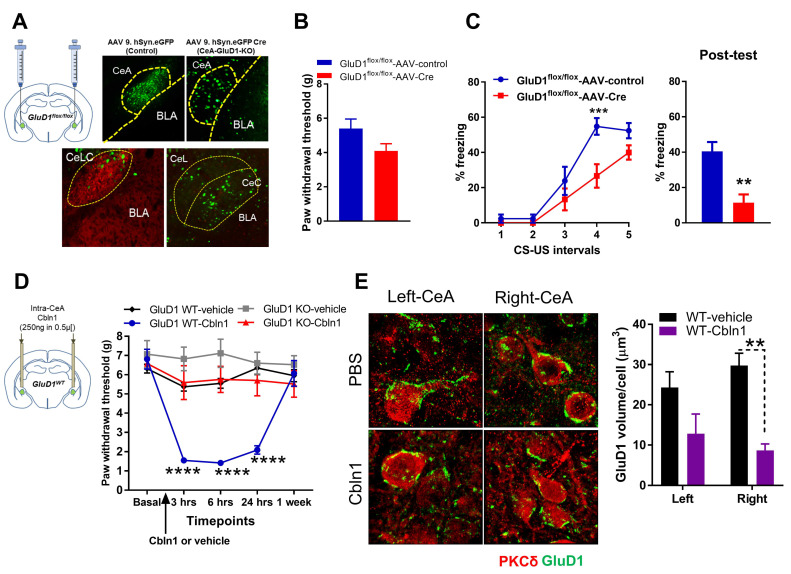
Recombinant Cbln1 in CeA increases mechanical hypersensitivity in normal animals. (**A**) Local deletion of GluD1 in the CeA using AAV constructs. Conditional deletion of GluD1 from CeA was achieved using Cre-lox strategy. Mice were injected with with AAV9-hsyn-eGFP or AAV9-hsyn-eGFP-Cre, bilaterally into CeA. Deletion of GluD1 from CeA was confirmed by immunohistochemistry. (**B**) Deletion of GluD1 from the CeA does not lead to significant change in mechanical hypersensitivity. (**C**) Impaired averse fear learning in mouse with GluD1 ablation in the CeA. Significant deficits in fear acquisition at 4th CS US (Two-way repeated measures ANOVA, F (1, 10) = 6.31, *p* = 0.031; Bonferroni’s post hoc test, 4th CS-US: AAV-control (*n* = 7 mice) vs. AAV-Cre (*n* = 5 mice): 54.764 ± 4.765 vs. 26.66 ± 6.665, *** *p* = 0.0008) as well as in retrieval 24 h later (AAV-control vs. AAV-Cre: 40.47 ± 5.282 vs. 11.46 ± 4.619, ** *p* < 0.005, Unpaired t-test with Welch correction). (**D**) Mice underwent cannulation surgery for the implantation of bilateral CeA cannulas. Effect of recombinant Cbln1 (250 ng in 0.5 µL) injected into CeA of naïve mice. After measuring basal mechanical hypersensitivity, Cbln1 was administered intracranially into the CeA and mechanical sensitivity (von Frey test) was measured in the right hind paw at 3 h, 6 h, 24 h, and 1 week. Significant increase in mechanical sensitivity is observed from 3 h to 24 h. (Two-way repeated measures ANOVA, treatment F (3, 15) = 8.77, *p* = 0.0009; Bonferroni’s post hoc test, WT-vehicle (*n* = 4 mice) vs. WT- Cbln1 (*n* = 5 mice): 3 h, 5.371 ± 0.223 vs. 1.553 ± 0.110, **** *p* < 0.0001; 6 h, 5.549 ± 0.253 vs. 1.418 ± 0.114, **** *p* < 0.0001; 24 h, 6.347 ± 0.341 vs. 2.088 ± 0.217, **** *p* < 0.0001). No change was seen in GluD1 KO mice after Cbln1 administration (*n* = vehicle (5 mice), Cbln1 (5 mice)). (**E**) Recombinant Cbln1 led to a significant reduction of perisomatic GluD1 at 6 h timepoint predominantly in right CeA compared to vehicle (PBS) injected mice. (Two-way ANOVA treatment F (1, 10) = 19.45, *p* = 0.0013; Bonferroni’s post hoc test, WT-vehicle (3 mice) vs. WT-Cbln1 (4 mice): Right CeA: 29.74 ± 3.08 vs. 8.72 ± 1.57, ** *p* = 0.0048; Left CeA: 24.31 ± 3.91 vs. 12.82 ± 4.89, *p* = 0.10).

To more precisely address the role of GluD1 in the CeA and overcome compensatory effects of deletion of GluD1 during development, we conducted local deletion of GluD1 using adeno-associated viral strategy (Figure 8A). AAV-control and AAV-Cre were injected into the CeA of GluD1^flox/flox^ mice and the effect on mechanical hypersensitivity was examined. No change in basal mechanical sensitivity was observed in these mice (Figure 8B). However, mice with ablation of GluD1 from CeA demonstrated impairment in averse learning in the cued fear conditioning paradigm (Figure 8C). We further examined whether Cbln1 may serve a physiological role in nociception. Administration of Cbln1 into the CeA in normal wildtype animals increased mechanical hypersensitivity (Figure 8D). This effect of Cbln1 was dependent on GluD1 as no change in sensitivity was observed in GluD1 KO. Increased hypersensitivity by Cbln1 lasted for at least 2 days and reached baseline by 1 week, suggesting a relatively long-lasting effect of Cbln1 injection. Cbln1 also induced place aversion in conditioned place preference testing (Supplementary Figure S6). We further addressed the potential relationship between Cbln1 injection on GluD1 expression. Interestingly, Cbln1 injection led to downregulation of GluD1 in the right CeA (Figure 8E), thus producing similar changes as in CFA and SNL models. These results suggest that Cbln1 release in the CeA may facilitate nociceptive inputs/processing and may serve as a teaching signal in avoidance learning.

## 4. Discussion

The amygdala and its central nucleus has been identified as a key brain structure important for both the nocifensive and averse-affective behaviors in various pain conditions. Pain-related neuroplasticity involving glutamatergic and neuropeptide systems has been observed consistently [7,8,9,10,11,13,14,16,17,18,19,20]. More recently, cell type–specific details of the CeA circuitry and their roles in nociception have been emerging [19,28,29,31]. Our results reveal a novel structural mechanism mediated by the GluD1-Cbln1 trans-synaptic signaling complex in the functional integrity of the PB-CeLC pathway; its dysregulation was found to underlie pain-related behaviors. The PB-CeLC synapses are structurally unique in that they have basket-like perisomatic organization in addition to the punctate elements. We found that GluD1 expresses as perisomatic structures in the CeLC and is absent in CeM. Consistent with the expression of the GluD1′s tripartite partner Cbln1 in PB neurons, GluD1 was found to localize preferentially at vGluT2 terminals which primarily arise from PB as opposed to vGluT1 terminals which primarily arise from BLA [57]. Moreover, GluD1 localized postsynaptically to CGRP terminals, which are known to arise from PB. In addition to input specificity, GluD1 also showed cell-type specificity with expression in PKCδ^+^ neurons compared to SOM^+^ neurons. The perisomatic and punctate elements of GluD1 were found on cell soma and punctate elements of PKCδ^+^ neurons. Previous studies have demonstrated that the GluDs have important roles in the organization of postsynaptic proteins via their C-terminal interactions [62,63]. Thus, the input- and cell-type specificity of GluD1 may impart unique characteristics to these synapses relevant to normal physiology as well as pain condition.

In both inflammatory and neuropathic pain conditions, we found a reduction in the perisomatic as well as punctate GluD1. The reduced expression was observed at 6 h, the earliest time point tested, and sustained for 1 week in the inflammatory pain model. The reduction in GluD1 was more prominent in the right CeLC and the reduction was strong at the 24 h time point and sustained thereafter. In contrast, reduction in Cbln1 puncta co-localized with GluD1 steadily declined over the 1-week period. The downregulation of GluD1 was also observed in a neuropathic pain model and was more prominent in the right CeLC. In contrast, AMPA receptor subunit GluA1 increased in both inflammatory and neuropathic pain models consistent with previous findings of neuroplasticity of PB-CeLC pathway [14]. Pain related changes in molecules regulating synapse structure has not been studied previously in the amygdala, but in the spinal cord, pain related changes in neurexin and neuroligin complexes have been observed [64,65,66]. An increase in these molecules is observed in inflammatory and neuropathic pain. It has previously been shown that there is a switch in the neurexin splice variant in response to synaptic activity [67,68]. Specifically, an increase in synaptic activity was found to reduce the S4^+^ splice variant which interacts with Cbln1 [36,38]. Thus, it is conceivable that pain related changes in the neurexin splice variants may contribute to changes in Cbln1 and GluD1 expression.

We found that a single intra-CeA injection of recombinant Cbln1 reversed mechanical hypersensitivity in the inflammatory pain model. This effect had a time course of several days and the reversal persisted for at least 1 week. In the GluD1 KO, Cbln1 was ineffective in reversing mechanical hypersensitivity, demonstrating a requirement for GluD1-Cbln1 signaling in this behavioral effect. In addition to Cbln1, GluD1 can also bind to other Cblns [69] of which Cbln2 and Cbln4 are enriched in the forebrain [44,70]. We examined whether Cbln2 could substitute for Cbln1 and produce antihyperalgesic effects and found that Cbln2 did not produce similar effects as Cbln1. This could be in part due to the lower affinity of Cbln2 for GluD1 [69]. Consistent with the lateralization of downregulation of GluD1, effects of recombinant Cbln1 on mechanical hypersensitivity in the inflammatory pain model showed right side lateralization. Furthermore, injection of recombinant Cbln1 into the right CeA was effective in mitigating pain-related audible and ultrasonic vocalizations, mechanical hypersensitivity and anxiety-like behavior in a neuropathic pain model. Observation of right hemispheric lateralization in the CeA has been reported in pain models [51,52,53] and the lateralization is thought to be independent of PB activation since it occurs bilaterally in the PB and neurochemical activation of PB is not correlated with CeA activation [71]. Our findings point to a potential molecular correlate for lateralization with GluD1 downregulation being more prominently observed in the right CeA and rescue by Cbln1 mediated by the right CeA. Together these studies demonstrate a very specific role of GluD1-Cbln1 signaling in the right CeA in pain-related aversive and affective behaviors.

An important observation in our studies is the slow but persistent time course of rescue of pain behaviors by recombinant Cbln1 in both inflammatory and neuropathic pain models. This time course supports a model in which recombinant Cbln1 normalizes the synapse structure rather than producing an acute effect. We found that exogenous Cbln1 restored the downregulated GluD1 and the upregulated GluA1 in both inflammatory and neuropathic pain models. This finding together with the absence of rescue of mechanical hypersensitivity by Cbln1 in GluD1 KO mice demonstrates that Cbln1 acts by restoring GluD1 expression and downstream synaptic function. We also found that recombinant Cbln1 restored the hyperexcitability of CeC neurons in the neuropathic pain model, suggesting that normalization of GluD1 also restores intrinsic properties of CeC neurons, supporting a structural role and ability to organize postsynaptic proteins.

We also conducted studies to examine the effect of D-serine on the antihyperalgesic effect of Cbln1 in the inflammatory pain model. D-serine binds to GluD1 and GluD2 subunits and induces conformational changes in the receptor. However, this does not lead to typical ion channel currents via these receptors [32,33]. It has been found that D-serine-induced conformational changes in delta receptors are similar to AMPA receptor desensitization involving breakdown of dimer interface [72], which has the potential to impair Cbln1 interaction with the GluD amino terminal domain. Our data demonstrates that D-serine impairs antihyperalgesic effects of Cbln1 potentially by inducing conformational changes in GluD1, which negatively impact its interaction with Cbln1. Furthermore, D-serine is able to produce sustained impairment of Cbln1 action possibly by preventing the initial recruitment of Cbln1 to synapses hence downstream restoration of synapses. However, in addition to being a ligand for GluD1, D-serine is also a co-agonist of NMDA receptors. Therefore, future experiments using GluD1 constructs that lack the ability to bind D-serine would provide a deeper understanding of the role of D-serine in CeA in pain modulation.

Recently, the roles of PKCδ^+^ and SOM^+^ neurons were addressed in a neuropathic pain model [29]. In the pain state, hyperexcitability was noted specifically in the CeLC PKCδ^+^ neuron subtype [29]. Furthermore, in late firing and presumable PKCδ+ neurons an increase in PB-CeLC neurotransmission [19] and presynaptic release probability was noted [28]. In contrast, no change in the excitability [29], but a decrease in synaptic efficacy [28] was found in CeLC SOM^+^ neurons. Chemogenetic inhibition of PKCδ^+^ neurons mitigated pain behaviors, whereas activation of these neurons produced pain-like behaviors in normal animals [29]. In agreement with these findings, we found that inhibition of PKCδ^+^ neurons mitigated mechanical hypersensitivity in an inflammatory pain model. Given the preferential expression of GluD1 in PKCδ^+^ neurons and the role of these neurons in pain modulation, we predict that the robust effect of recombinant Cbln1 on pain behaviors may arise due to cell-specific actions.

We also evaluated pain behaviors in GluD1 KO. No difference in basal pain sensitivity was observed in these models. GluD1 KO mice also did not exhibit differences in CFA-induced mechanical hypersensitivity, suggesting that GluD1 is not critical for the acute stage or induction of pain. Instead, the loss of antihyperalgesic effects of Cbln1 in GluD1 KO suggests a requirement of GluD1 for recovery from pain progression. We also found that GluD1-Cbln1 signaling in the CeA is critical for averse learning. Deletion of GluD1 from CeA reduced fear learning, which is consistent with the fear-conditioning deficit observed in GluD1 KO mice [46]. In addition, conditional deletion of Cbln1 also results in a fear-learning deficit [45]. Thus, Cbln1 release and binding to GluD1 may be necessary to initiate fear learning. Interestingly, injection of recombinant Cbln1 in the CeA induced mechanical hypersensitivity in normal animals, which was absent in GluD1 KO. Intra-CeA Cbln1 also induced place aversion behavior and led to a downregulation of GluD1 similar to changes observed in pain models. Thus, GluD1-Cbln1 complex initiates a unique structural signaling in normal animals that may lead to bidirectional changes in postsynaptic composition, neuronal responsiveness, and behavior. Our studies have identified recombinant Cbln1 and GluD1-Cbln1 signaling as a novel mechanism of pain-related amygdala plasticity that can be targeted to restore synaptic function in chronic pain. With our growing understanding of protein therapeutics and biologics, this finding may have important clinical implications.

## Data Availability

Data is available from the corresponding author upon reasonable request.

## Supplementary Materials

The following are available online at https://www.mdpi.com/article/10.3390/cells10102644/s1, Figure S1: 3D reconstruction video of GluD1 (green) localization on PKCδ^+^ neurons (red) in the CeA; Figure S2: GluD1 effect on excitatory neurotransmission and excitability. Figure S3: Time course of GluD1 and Cbln1 downregulation in CFA-induced pain model; Figure S4: Effect of recombinant Cbln1 in pain models; Figure S5: Deletion of GluD1 does not affect basal pain sensitivity; Figure S6: Intra-CeA Cbln1 leads to conditioned place aversion in mice; Figure S7: Western blot original images.
