# Supplementary material for: Dysfunction of Glutamate Delta-1 Receptor-Cerebellin 1 Trans-Synaptic Signaling in the Central Amygdala in Chronic Pain
Source: Cells. 2021 Oct 3;10(10):2644. doi: 10.3390/cells10102644 (PMC8534524; doi:10.3390/cells10102644)

EXTENDED DATA:

Supplementary Figure S1. (A) 3D reconstruction video of GluD1 (green) localization on PKCδ+ neurons (red) in the CeA.

Supplementary Figure S2. GluD1 effect on excitatory neurotransmission and excitability. (A) No significant effect of GluD1 deletion on excitatory neurotransmission in CeL neurons (Frequency: $p = 0.16$, Amplitude: $p = 0.74$, two-tailed unpaired t-test, df = 14, n = 8). **(B)** No significant change in excitability of CeC neurons in GluD1 KO mice (Two-way repeated measures ANOVA, genotype F (1, 27) = 2.61, $p = 0.12$; WT: n = 14, GluD1 KO: n = 15).

Supplementary Figure S3. Time course of GluD1 and Cbln1 downregulation in CFA-induced pain model. (A) Immunohistochemistry analysis indicating the trajectory of GluD1 volume reduction around PKCδ cell at 6, 24, 48 hrs and 1-week timepoints after intraplantar CFA administration (One-way ANOVA, treatment F (4, 20) = 20.1, $p < 0.0001$; Bonferroni's post hoc test, Saline (n = 8 mice) vs. CFA 6 hrs (n = 3 mice) *$p = 0.019$, vs CFA 24 hrs (n = 3 mice) ***$p = 0.0001$, vs CFA 48 hrs (n = 6 mice) ****$p < 0.0001$, vs CFA-1 week (n = 5 mice) ****$p < 0.0001$). **(B)** Immunohistochemistry analysis indicating the trajectory of Cbln1 puncta number reduction at 6, 24, 48 hrs and 1-week timepoints after intraplantar CFA administration (One-way ANOVA, treatment F (3, 8) = 34.85, $p < 0.0001$; Bonferroni's post hoc test, Saline (n = 3 mice) vs. CFA 6 hrs (n = 3 mice) **$p = 0.004$, vs CFA 24 hrs (n = 3 mice) ***$p = 0.0004$, vs CFA-1 week (n = 3 mice) ****$p < 0.0001$). **(C)** Western blot analysis showing downregulation of GluD1 in synaptoneurosomal preparation of CeA. Induction of inflammatory pain lead to a downregulation of GluD1 in the CeA at 48 h and 1 week after CFA injection (One-way ANOVA, treatment F (2, 17) = 6.35, $p = 0.0087$, Dunnett's multiple comparison, Saline (n = 7 mice) vs. CFA 48 hrs (n = 7 mice): 1.02 ± 0.05 vs. 0.69 ± 0.08, **$p = 0.0094$; Saline vs CFA 1 week (n = 6 mice): 1.02 ± 0.05 vs. 0.71 ± 0.08, *$p = 0.019$). **(D)** An upregulation of AMPA receptors is observed in the model of inflammatory pain in the CeA 1 week

after CFA injection (Saline vs. CFA: 1 week time point- 0.975 ± 0.032 vs. 1.169 ± 0.066, *p = 0.0401, Unpaired t-test, n = 4 mice/treatment).

Supplementary Figure S4. Effect of recombinant Cbln1 in pain models. (**A**) Effect of recombinant Cbln1 injected at a lower volume (250 ng in 250 nl on each side) in the CeA also rescued mechanical hypersensitivity (n = 3 mice). Injection of recombinant Cbln1 (250 ng in 250 nl on each side) into the dorsal striatum (DS) did not lead to a persistent reduction in mechanical hypersensitivity in von Frey test (n = 3 mice). No effect of Cbln1 in the dorsal striatum in GluD1 KO (n = 3 mice). (**B**) Cbln1 reduced mechanical hypersensitivity in cisplatin-induced neuropathic pain model. Intracerebroventricular injection of Cbln1 (1.5 µg and 3 µg) reduced mechanical hypersensitivity in cisplatin-induced neuropathy mice (Two-way repeated measures ANOVA (6 hrs -96 hrs after each injection) Treatment F (1, 7) = 5.87, p = 0.046, n = 4 mice (vehicle), 5 mice (Cbln1)).

Supplementary Figure S5. Deletion of GluD1 does not affect basal pain sensitivity. Wildtype and GluD1 KO were evaluated for **(A)** tail flick test, **(B)** hot plate test, **(C)** von Frey filament and **(D)** gabapentin-induced conditioned place preference tests. No changes these paradigms were observed (n=6-8 mice). **(E)** Sensitivity in formalin test was assessed in wildtype and GluD1 KO. Significantly higher licking behavior was observed in GluD1 KO (Two-way ANOVA, interaction F (1, 21) = 6.25, p = 0.020, Bonferroni's post hoc test, WT formalin (n = 8 mice) vs GluD1 KO formalin (n = 5 mice), *p = 0.033, WT saline (n = 7 mice), GluD1 KO saline (n = 5 mice)).

Supplementary Figure S6. (A) Intra-CeA Cbln1 leads to conditioned place aversion in mice (Vehicle (n= 4 mice) vs Cbln1 (n = 6 mice), **-** 7.925 ± 4.868 vs. – 67.38 ± 21.97, *p = 0.0419, unpaired t-test).

Supplementary Figure S7. Western blot original images.

Supplementary Figure S1 Video

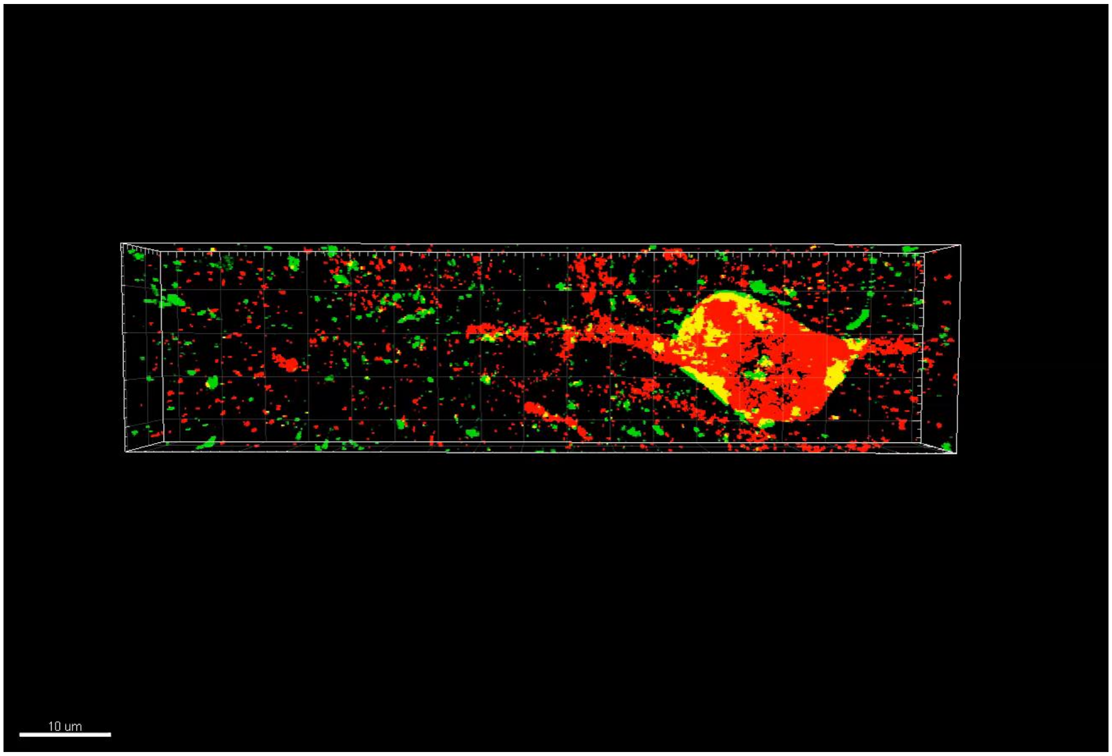

Supplementary Figure S2

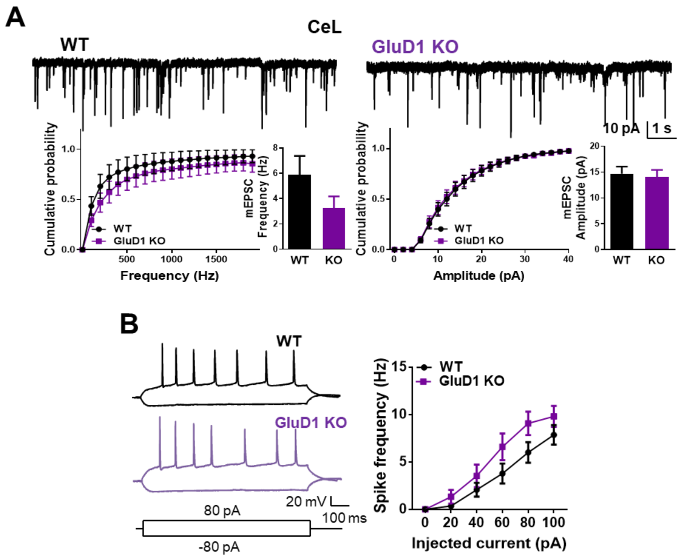

Supplementary Figure S3

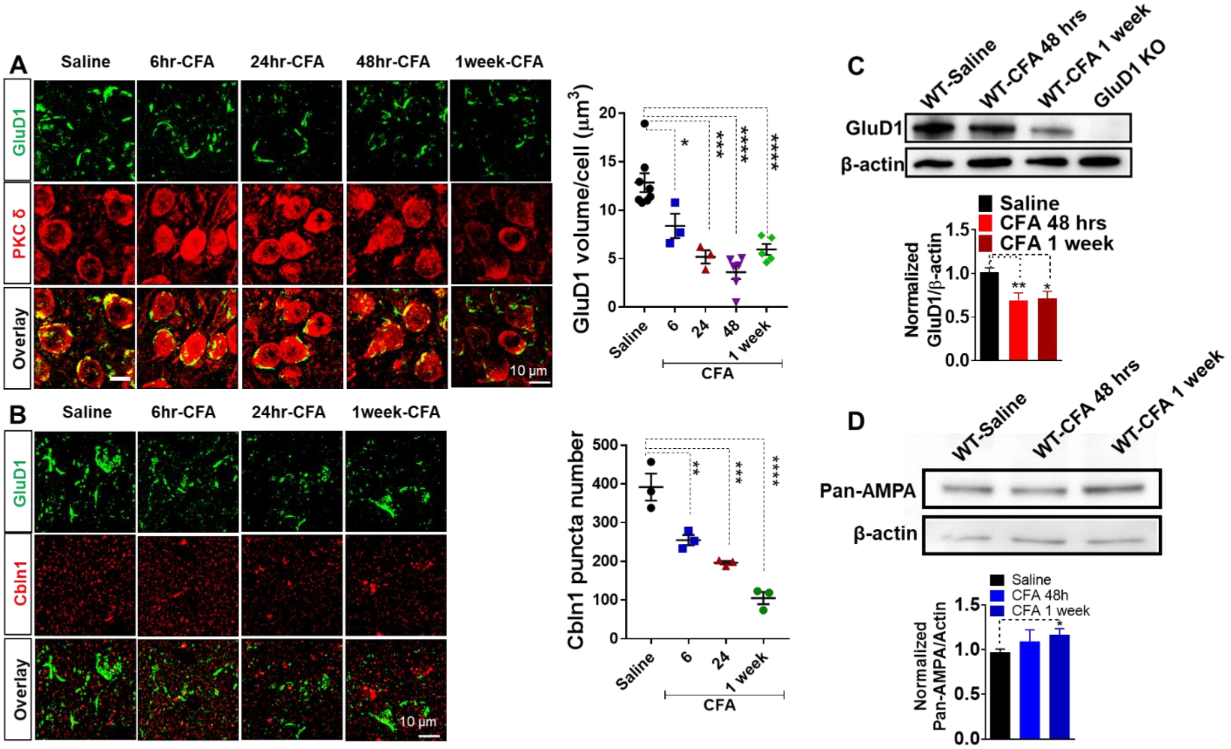

Supplementary Figure S4

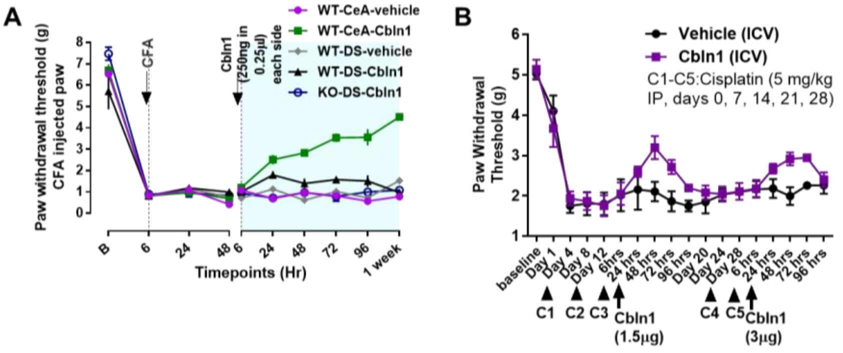

Supplementary Figure S5

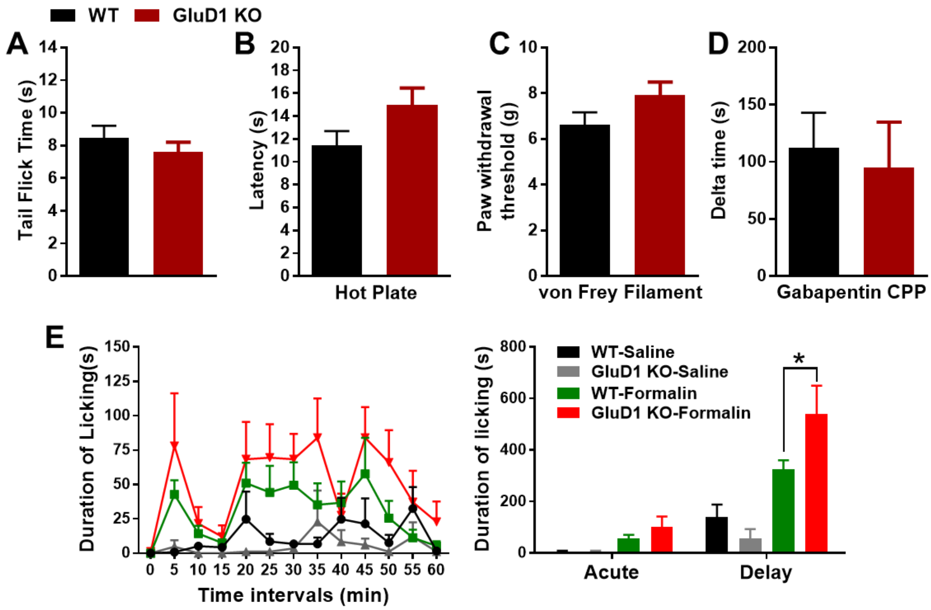

Supplementary Figure S6

A

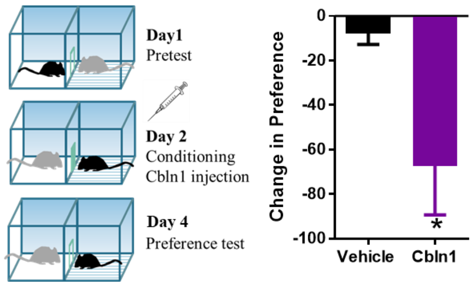

Supplementary Figure S7: Western blot original images.

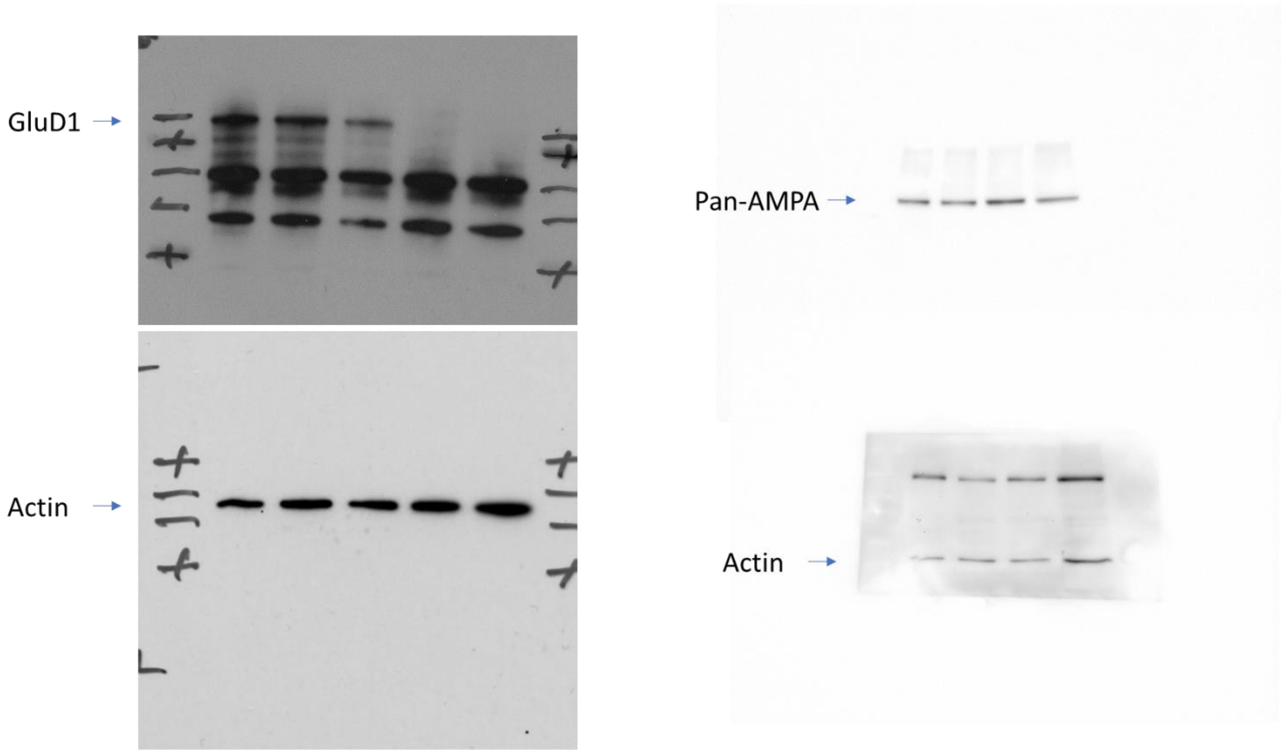

Supplement: Supplementary file 1 [file cells-10-02644-s001.zip › cells-1394050 SM Final copy 2.pdf]
